# Green synthesis of nitrogen-doped self-assembled porous carbon-metal oxide composite towards energy and environmental applications

**DOI:** 10.1038/s41598-019-41700-5

**Published:** 2019-03-26

**Authors:** Arpita Ghosh, Sreetama Ghosh, Garapati Meenakshi Seshadhri, Sundara Ramaprabhu

**Affiliations:** Alternative Energy and Nanotechnology Laboratory (AENL), Nano Functional Materials Technology Center (NFMTC), Department of Physics, Indian Institute of Technology Madras, Chennai, 600036 India

## Abstract

Increasing environmental pollution, shortage of efficient energy conversion and storage devices and the depletion of fossil fuels have triggered the research community to look for advanced multifunctional materials suitable for different energy-related applications. Herein, we have discussed a novel and facile synthesis mechanism of such a carbon-based nanocomposite along with its energy and environmental applications. In this present work, nitrogen-doped carbon self-assembled into ordered mesoporous structure has been synthesized via an economical and environment-friendly route and its pore generating mechanism depending on the hydrogen bonding interaction has been highlighted. Incorporation of metal oxide nanoparticles in the porous carbon network has significantly improved CO_2_ adsorption and lithium storage capacity along with an improvement in the catalytic activity towards Oxygen Reduction Reaction (ORR). Thus our present study unveils a multifunctional material that can be used in three different fields without further modifications.

## Introduction

The increasing consumption of fossil fuels has largely enhanced the accumulation of CO_2_ in the atmosphere. This has led to different global challenges like environmental pollution, greenhouse effect, and energy shortage. In this regard, there is an urgent need for materials that can capture this greenhouse gas and can be used in energy-related applications, all in one. Conventional amine scrubbing process for capturing CO_2_ has several disadvantages like high regeneration cost, extensive corrosion, and loss of amines during their generation process^1^. So novel porous materials having high CO_2_ adsorption capacity attracted considerable attention in recent times mainly because of their low cost, ease of synthesis, wide availability, good chemical stability, and high specific surface area^2,3^. As an alternative of fossil fuel and to mitigate the increasing CO_2_ concentration in the atmosphere, renewable alternative energy storage and conversion technologies like battery and fuel cell have also been thought of as a part of this work. Rechargeable lithium-ion batteries (LIBs) are the most efficient and convenient electrochemical storage devices for portable as well as stationary applications. Along with the lithium storage capacity, an ideal anode material for LIB should possess high electrical conductivity and low volume expansion coefficient. On the other hand, Oxygen Reduction Reaction (ORR) is the most crucial cathode reaction in fuel cell and metal-air batteries to determine the efficiency and longevity of the device. To improve the sluggish reaction kinetics, platinum (Pt) and platinum-based alloy catalysts have been reported to show the best electrocatalytic activity. But, the high cost, less availability and poor durability of Pt and Pt-based alloys have led to the search for an alternative non-platinum based catalyst^4^.

Nitrogen doping also enhances the gas adsorption capacity by increasing the basic sites in the sample where CO_2_ gets anchored easily by Lewis acid (CO_2_) – Lewis base (N) interaction. The presence of basic nitrogen groups increase the interaction between the large quadrupole moment of CO_2_ molecules and the polar nitrogen sites^5^. The N-doped sites also help in adsorption of the oxygen molecule and act as active catalytic sites towards ORR^6^. Porous carbon materials with high specific surface area, tunable pore size and large pore volume have played a very vital role in all the above-mentioned applications.

Metal oxides, as well as hydroxides, serve as a good candidate for CO_2_ capture at moderate temperature due to their low cost and wide availability. Many experimental studies on high CO_2_ capture capability of metal oxides have already been reported. These metal oxides act as heterogeneous catalysts and so the interaction between CO_2_ and the oxide surfaces is of great interest^7,8^. Physicochemical adsorption of CO_2_ on iron oxide decorated graphene nanocomposite has already been reported by A. K. Mishra *et al*.^9^. Tamilarasan *et al*. had worked with polyaniline-magnetite nanocapsules showing high CO_2_ capture capability as well as fast sorption kinetics of the nanocomposite^10^. In the case of LIBs, the specific capacity of any graphitic carbon is limited by the theoretical capacity of these materials. So, incorporation of any metal/metal oxides, that can take part in conversion type redox reaction, improves the specific capacity considerably^11,12^. In this regard, combining the two storage mechanisms (i.e. insertion/de-insertion and conversion) using a single material can be found as an efficient way to enhance the storage capacity of LIBs. Fe_3_O_4_ incorporated nitrogen doped porous carbon has been extensively studied as an efficient anode material for LIBs^13–16^. Iron-oxide nanoparticles were chosen to be a superior catalyst owing to their low cost, high stability and intrinsic catalytic activity towards ORR in alkaline medium.

In this present work, we have synthesized metal oxide-porous carbon composite by simple self-assembly and coprecipitation techniques. The pore-foaming agent used in forming the porous carbon framework is sodium hydrogen carbonate that is nontoxic and cheap. Briefly, the pore formation mechanism based on hydrogen bonding interactions has also been explained here. Further, this particular nanocomposite has been explored for CO_2_ capture, lithium-ion battery and ORR catalyst in alkaline fuel cell. It has been investigated that this nanocomposite has the potential to serve as an effective CO_2_ adsorbent, anode material for lithium-ion battery and efficient ORR catalyst for fuel cell operations because of its interconnected porous network, high nitrogen doping and presence of metal oxide nanoparticles (Fe_3_O_4_) on its surface. In the case of lithium-ion battery, dynamic electrochemical spectroscopy (DEIS) measurement has been carried out in order to estimate the actual potential window of the significant electrochemical reactions involved in it. In order to address the burning environmental challenges associated with increasing concentration of greenhouse gases and a shortage of renewable energy storage and conversion devices, our approach is the first of its kind where a single environment-friendly composite can be used in three above-mentioned fields.

## Results and Discussion

**Figure 1:**
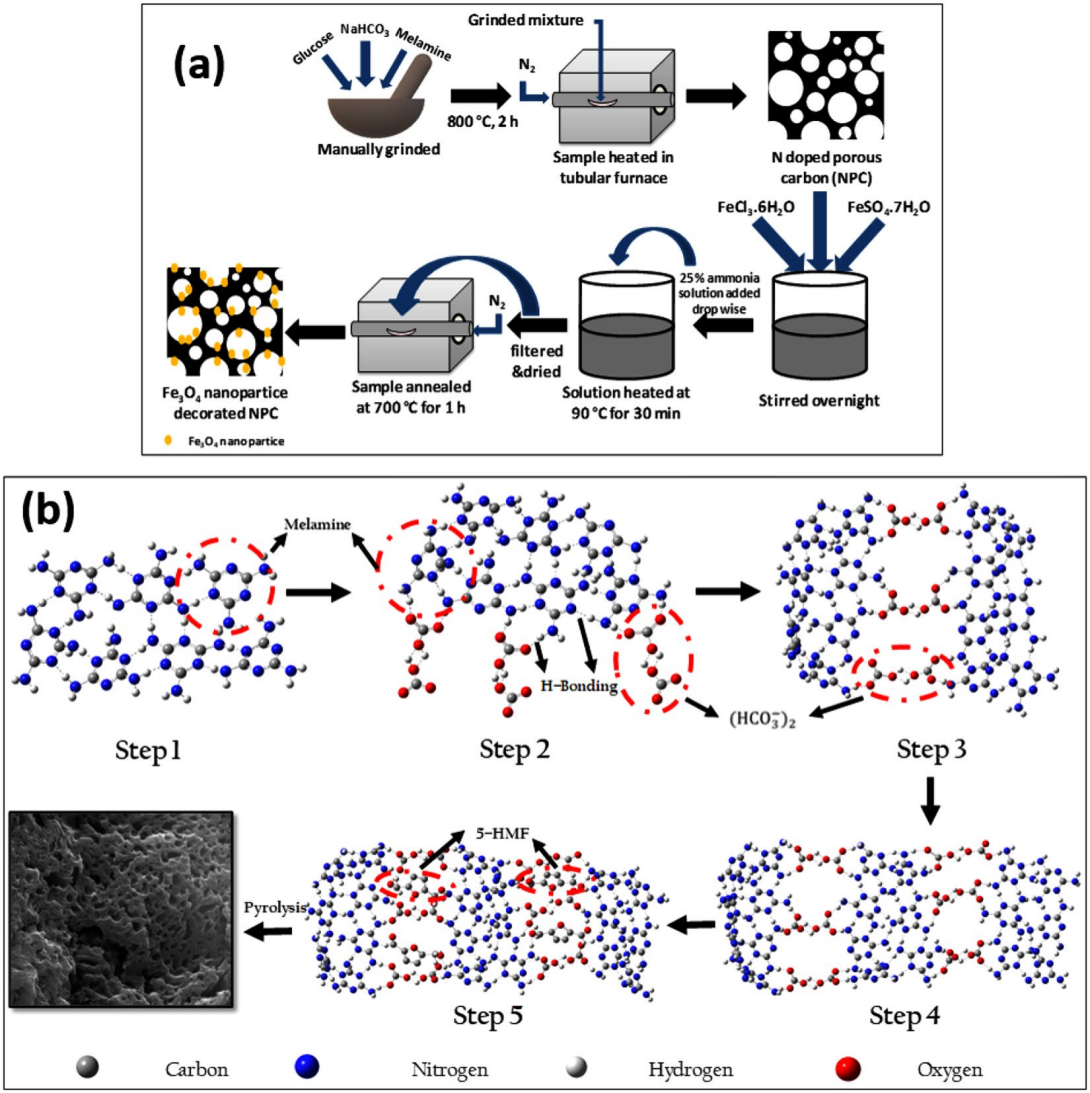
(**a**) Synthesis of Fe_3_O_4_ nanoparticles decorated N doped porous carbon composite and (**b**) Pore formation mechanism in NPC.

The coprecipitation technique, used to synthesize iron oxide, is a simple and efficient chemical pathway where aging of a stoichiometric mixture of ferrous and ferric salts in aqueous medium takes place^17^. Kim *et al*. explained in details the formation pathway of magnetite nanoparticles by coprecipitation method. In typical coprecipitation technique, as the ammonia solution is added to the iron salt solution, interfacial contact forms in between the iron-rich solution (pH ~ 1.5) and the base (pH ~ 11). So diffusion occurs in both ways: the base tries to diffuse into the iron-rich solution while iron ions try to diffuse in the opposite direction. So precipitation occurs at the interface. The authors have mentioned that during the reaction mechanism many intermediate products are formed. As the two solutions begin to mix, two types of nucleation process will be initiated simultaneously. On one hand, the low pH iron side increases the pH by reacting with ammonia solution which basically reacts with the less stable Fe^3+^ ions to form the following intermediates: akaganeite → goethite → (hematite → maghemite) → magnetite. On the other hand, ammonia reacts with both Fe^2+^ as well as Fe^3+^ to form ferrous hydroxide → lepidocrocite → (maghemite) → magnetite^17^. This briefly explains the formation pathway of magnetite nanoparticles by coprecipitation method. This particular method explains a wide particle size distribution of the synthesized nanoparticles. The coprecipitation method is a very eco-friendly method for producing magnetite nanoparticles. It neither produces any toxic intermediates nor requires any complex precursors. However, controlling the particle size distribution is difficult in this process because the growth of the crystal depends only on kinetic factors^17^. The detailed synthesis procedure has been shown in Fig. 1(a).

### Pore formation mechanism in NPC

The pore formation mechanism in NPC is explained in Fig. 1(b). Dependence of pore formation on hydrogen bond interaction plays a key role in determining the morphology of the porous carbon. Herein, a probable mechanism of pore formation in highly porous carbon framework has been proposed which involves self-assembly of the precursor molecule (melamine) via hydrogen bonding interaction. Melamine molecules are linked via N–H∙∙∙N hydrogen bonding in order to form a crumpled planar sheet-like host lattice framework (Step 1). These sheets are further alternatively linked by (HCO_3_^−^)_2_ anions units (highlighted by red circle) originating from the bicarbonate salt precursor via N–H∙∙∙O bond formation (Step 2 and 3) to form an anionic host lattice with the interconnected open network (Step 4)^18^. Sodium bicarbonate promotes self-assembly among the precursors before pyrolysis that is beneficial for the synthesis of porous carbon^19^. It is well reported that glucose can be transformed into 5-hydroxy-methylfurfural (5-HMF) under thermal decomposition^20^. Thus introducing glucose in this anionic host lattice framework results in the formation of N–H∙∙∙O and C–H∙∙∙N hydrogen bonding in between 5-HMF and the host (highlighted by the red circle) (Step 5). Finally, during pyrolysis, melamine and sodium bicarbonate decomposed and 5-HMF dehydrated to form the porous network. Evolution of water molecules from 5-HMF and carbon dioxide from the decomposition of sodium bicarbonate has led to the formation of the interconnected mesopores in the host lattice.

**Figure 2:**
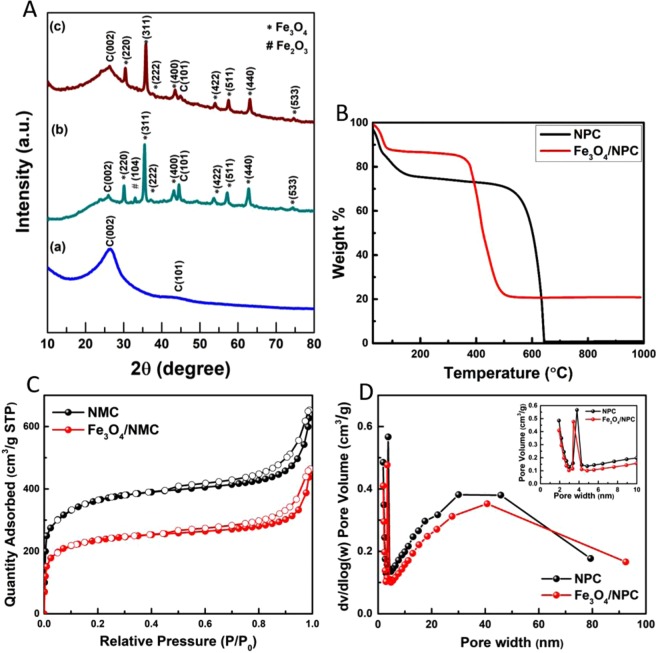
(**A**) XRD pattern of (a) Nitrogen-doped porous carbon (NPC), (b) Fe_3_O_4_/NPC before annealing and (c) Fe_3_O_4_/NPC after annealing and (**B**) Thermogravimetric analysis (**C**) Nitrogen adsorption (filled symbols)/desorption (empty symbols) isotherms at 77 K and (**D**) BJH pore-size distribution curve (inset enlarged view up to 10 nm) of NPC and Fe_3_O_4_/NPC respectively.

Fig. 2A(a) represents the XRD pattern of nitrogen-doped porous carbon (NPC). The diffraction peaks at 26.4° (002) and 44.8° (101) occur due to the graphitization of porous carbon. Prior to annealing, the as-synthesized Fe_3_O_4_/NPC (Fig. 2A(b)) shows a very less intense Fe_2_O_3_ peak at 33° (104) which disappears after annealing at 700 °C. Figure 2A(c) shows the XRD pattern of the porous carbon with pure Fe_3_O_4_ phase having a cubic spinel structure^21^. Thermogravimetric Analysis (TGA) of Fe_3_O_4_/NPC was carried out in air atmosphere from room temperature up to 800 °C at a heating rate of 20 °C min^−1^ (Fig. 2B) (details in SI).

**Figure 3:**
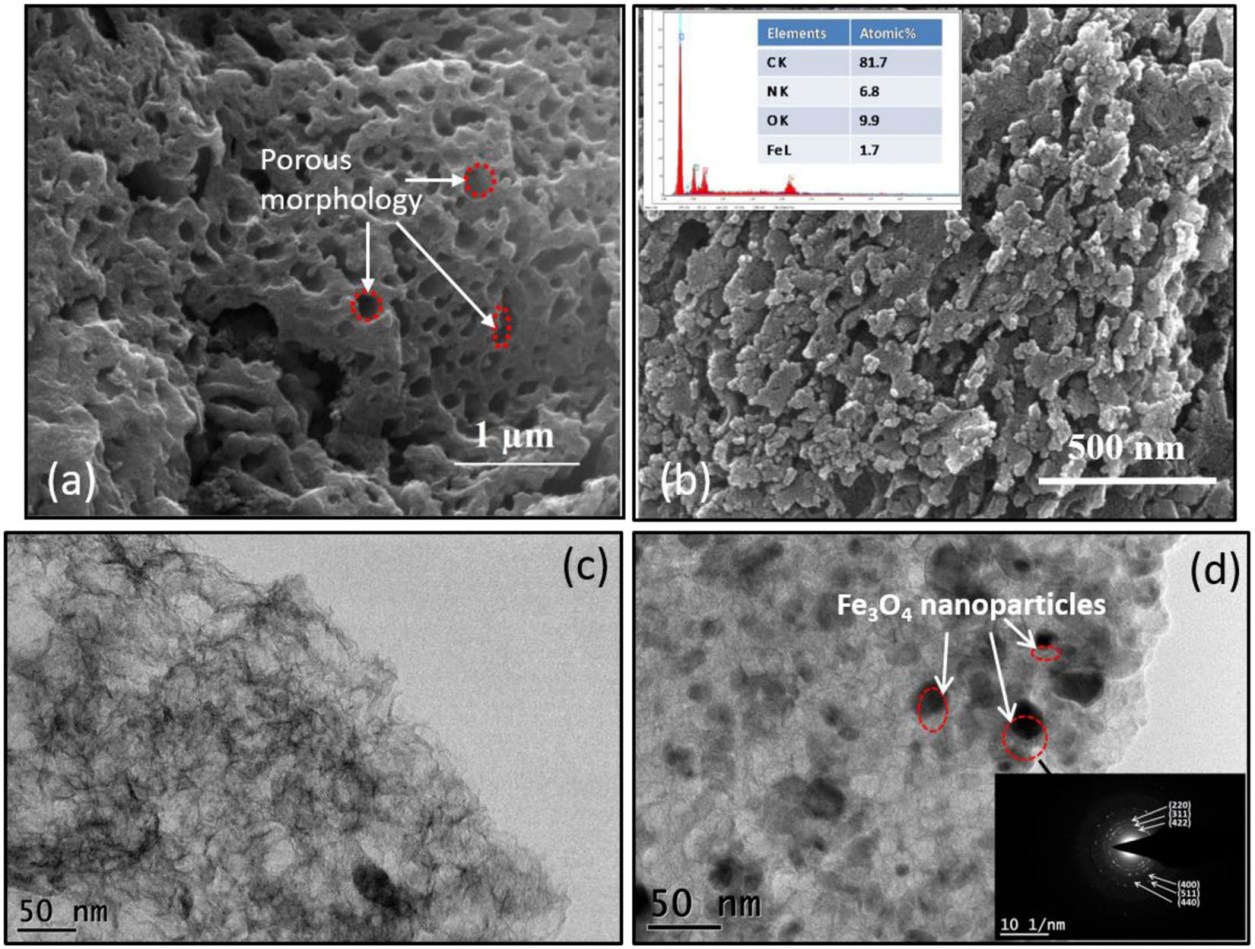
FESEM (**a** and **b**) (inset EDAX analysis) and HRTEM (**c** and **d**) (inset SAED pattern) images of NPC and Fe_3_O_4_/NPC respectively.

Gas adsorption as well as battery and fuel cell performance largely depend on the specific surface area and the pore size distribution of the nanocomposite. Fig. 2C shows the nitrogen adsorption/desorption isotherms at 77 K of the as-prepared samples. Both the samples exhibit type IV isotherms having a hysteresis loop that is consistent with the mesoporous nature of the samples. At low relative pressure region (P/P_0_ < 0.1), the curves show a high N_2_ uptake suggesting that some microporous nature is also present in the samples. The nitrogen uptake then becomes moderate at relatively intermediate pressures. As can be seen in Table 1, nitrogen doped porous carbon (NPC) showed a high specific surface area of 1182 m^2^ g^−1^ and a total pore volume of 1.01 cm^3^ g^−1^. Unlike N doped porous carbon (NPC), the surface area as well as the pore volume decreased but the average mesopore size slightly increased indicating a widening of the mesopores and decline of mesoporous ordering with the incorporation of Fe_3_O_4_ nanoparticles in the porous carbon framework^22^. The increase in pore size most likely might be due to the partial penetration of the nanoparticles leading to an expansion in the nanostructure. It might also be due to the enhanced rigidity caused by the Fe_3_O_4_ nanoparticles to decrease the pore shrinkage^23^. The increase in the size of the pores can be identified in the SEM and TEM images as well as shown in Fig. 3. The pore size distribution curve shows a peak centering around 3 nm (as shown in Fig. 2D inset) along with a broad peak in larger pore diameter region indicating mostly the presence of mesopores in the samples (Fig. 2D).

**Table 1:** Summary of the properties of as-grown samples (BET specific surface area, pore size, and pore volume).

Samples	S_BET_ (m^2^ g^−1^)	V_p_ (cm^3^ g^−1^)^a^	V_micro_ (cm^3^ g^−1^)^b^	V_meso_ (cm^3^ g^−1^)^c^	D_pore_ (nm)^d^
NPC	1182	1.01	0.308	0.720	3.42
Fe_3_O_4_/NPC	769	0.72	0.211	0.509	3.74

^a^Total pore volume (V_p_) obtained at P/P_0_ = 0.99; ^b^Micropore volume determined from t-plot; ^c^Mesopore volume determined by the difference between the total pore volume and the micropore volume; ^d^Average pore diameter (D_pore_).

The morphological studies of the prepared nanocomposite has been carried out using electron microscopy techniques. Fig. 3(a and b) shows the scanning electron microscopy (SEM) images of NPC and Fe_3_O_4_/NPC respectively. SEM image of the mesoporous carbon support material (NPC) shows the presence of innumerable pores in the carbon framework. The decoration of this porous framework with Fe_3_O_4_ nanoparticles can be seen in Fig. 3(b). The EDAX analysis (Fig. 3(b) inset) confirms the presence of C, N, O and Fe in the sample. The nitrogen content in the sample has been found to be around 7 at %, which suggests that the sample is highly N-doped. It can be seen here that the nanoparticles have not completely blocked all the pores in the porous carbon network. Fig. 3(c) shows the TEM image of the porous carbon, whereas, Fe_3_O_4_ nanoparticles decorated porous carbon is shown in Fig. 3(d) with the SAED pattern of Fe_3_O_4_/NPC shown in the inset. It suggests almost uniform distribution of Fe_3_O_4_ nanoparticles on the surface of the porous carbon framework. The size of the pores in Fe_3_O_4_/NPC is seen to be slightly larger than those of NPC. This is in confirmation with the results obtained from BET analysis.

**Figure 4:**
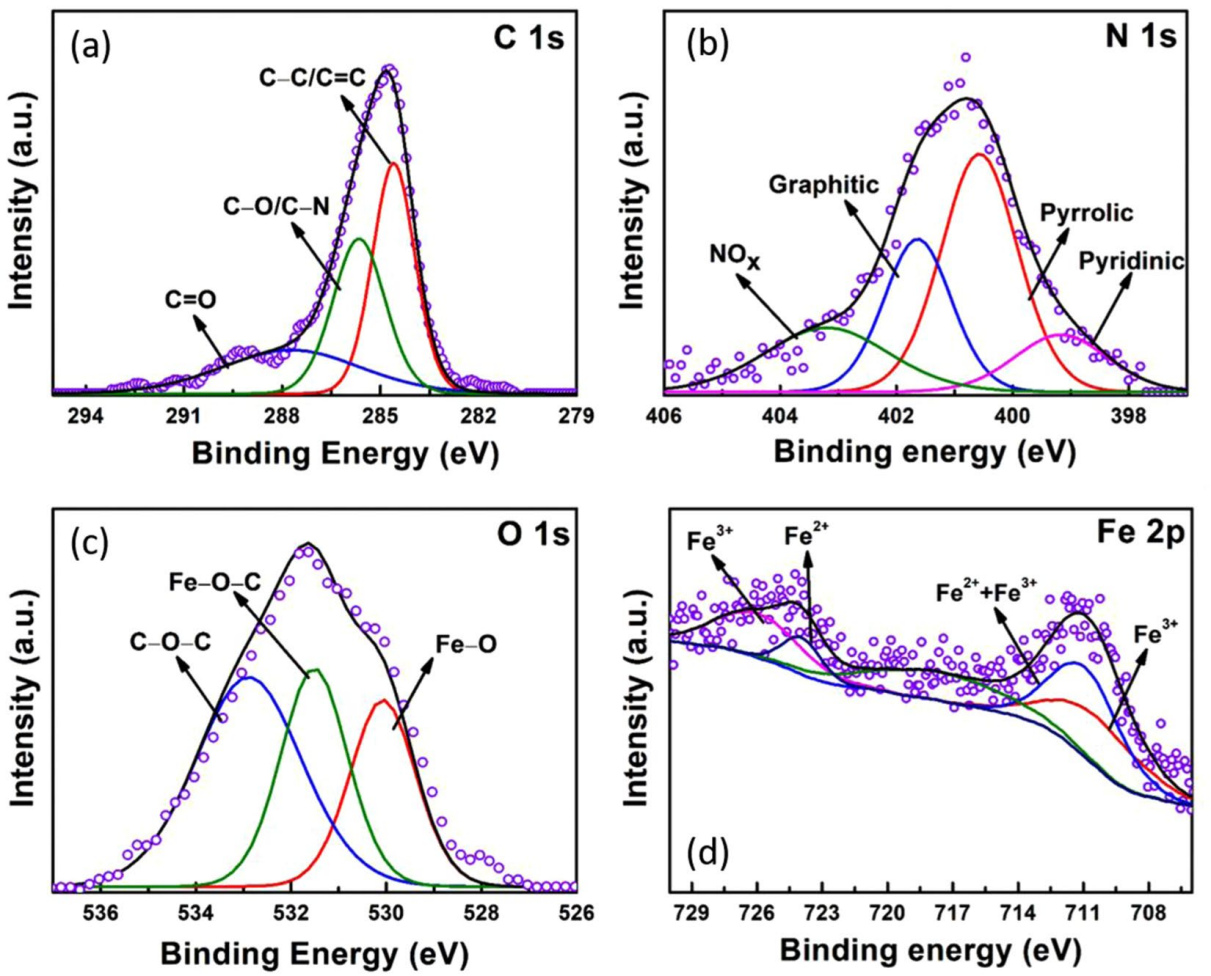
High resolution XPS spectra of (**a**) C 1 s, (**b**) N 1 s, (**c**) O 1 s, (**d**) Fe 2p of Fe_3_O_4_/NPC.

The high resolution XPS spectrum of C1s peak (Fig. 4(a)) is deconvoluted into three components with binding energies 284.7 eV, 285.9 eV, and 288 eV. These peaks are assigned to sp^2^ C=C, carbon-nitrogen complex C–N (sp^3^) and oxygenated carbon complex (CO_x_) respectively. N 1 s peak is deconvoluted into four components with binding energies 398.0 eV, 400.21 eV, 402.24 eV and 404.17 eV (Fig. 4(b)). The first three peaks are assigned to pyridinic, pyrrolic and graphitic nitrogen. A significant peak shift might have taken place because of the different environments of the N species^24^. Lastly, the low-intensity fourth peak corresponds to the π excitations resulting in positive charge accumulation on the N species situated at the edges^25^. The O 1 s spectrum is deconvoluted into three components (Fig. 4(c)) with binding energies 530.1 eV, 531.5 eV and 532.9 eV. The first peak corresponds to the anionic oxygen in Fe_3_O_4_^15,26^. The other two peaks indicate the existence of adsorbed atmospheric oxygen in the support material^27^. The high-resolution doublet of Fe 2p spectra (Fig. 4(d)) is deconvoluted into six peaks. Peaks located at binding energies of 724.4 eV is assigned as the Fe^2+^ oxidation state of Fe (2p_1/2_). The other peak at 710.6 eV signifies the coexistence of Fe^3+^ as well as Fe^2+^ of Fe (2p_3/2_)^28^. Other two peaks with binding energies 725.2 eV and 713.1 eV are allocated to the Fe^3+^ oxidation state of Fe (2p_1/2_) and Fe (2p_3/2_)^29^. The XPS spectra of both NPC and Fe_3_O_4_ before and after coupling are presented in Fig. S1 and compared.

### High-pressure CO_2_ adsorption studies

**Figure 5:**
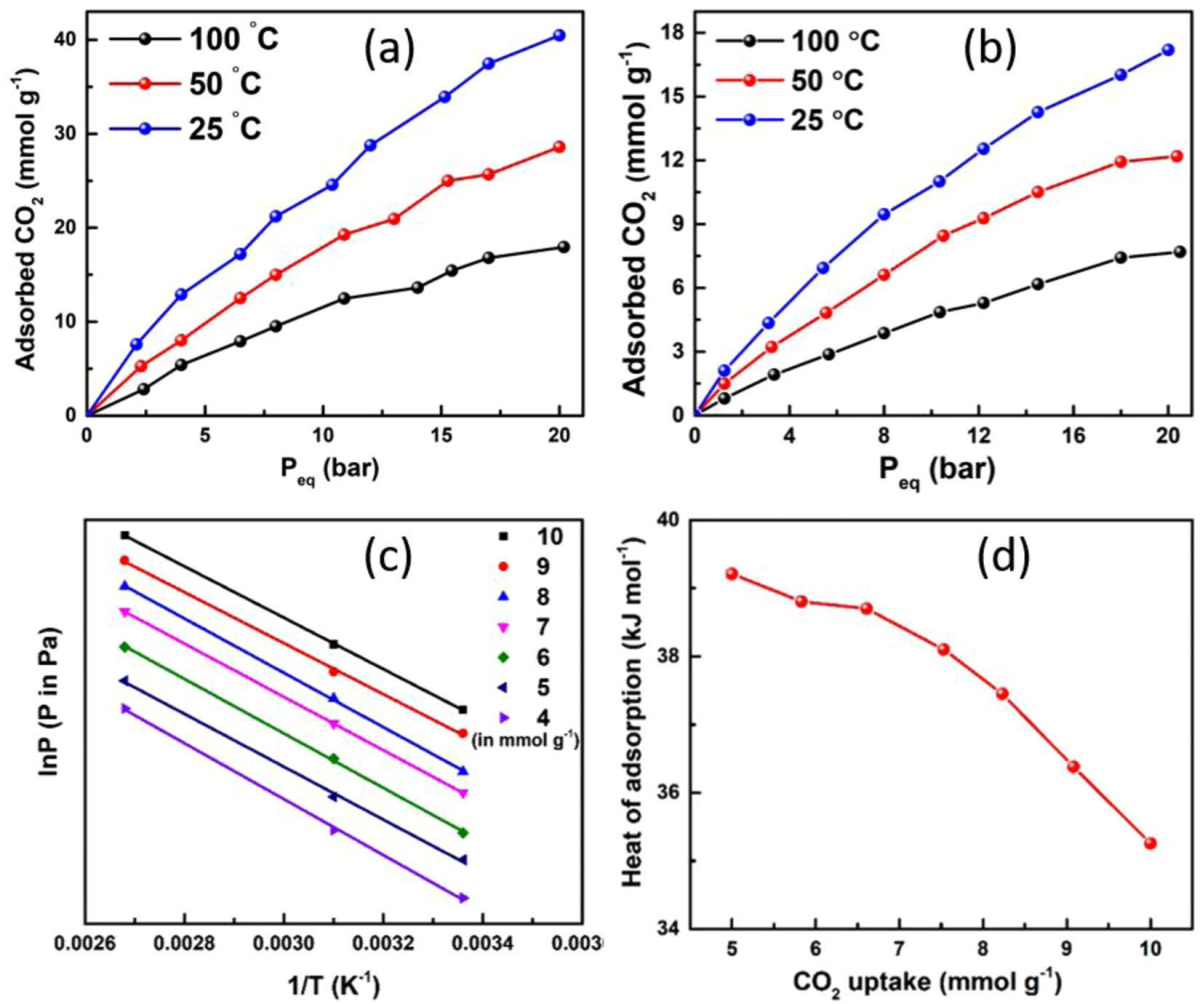
(**a**) CO_2_ adsorption isotherm of Fe_3_O_4_/NPC, (**b**) CO_2_ adsorption isotherm of NPC, (**c**) adsorption isosteres with different adsorbed amount of CO_2_ and (**d**) isosteric heat of adsorption of Fe_3_O_4_/NPC.

High-pressure CO_2_ adsorption studies have been carried on Fe_3_O_4_ decorated N doped porous carbon using Sievert’s apparatus using Pressure Swing adsorption technique. Fe_3_O_4_ nanoparticles are basic in nature and therefore Fe_3_O_4_/NPC consists of a large number of basic functional sites that attract acidic CO_2_ towards it and enhances adsorption. The nitrogen functionalities also play a significant role in the enhanced adsorptive behavior of the nanocomposite. Fe_3_O_4_/NPC has shown a high CO_2_ adsorption capacity of 40.5 mmol g^−1^ at 25 °C and at 20 bar equilibrium pressure in comparison to NPC which has shown a capacity of 17.2 mmol g^−1^ under the same conditions of temperature and pressure (Fig. 5(a and b)). The incorporation of metal oxide NPs has enhanced the adsorption capability of the support material (porous carbon) by more than two folds. Such high adsorption capacity can be attributed to the strong chemical interaction of the metal oxide with the CO_2_ gas forming some carbonate or bicarbonate species whereas physical adsorption of CO_2_ occurs in the porous carbon framework^9^. Thus, physicochemical adsorption of CO_2_ occurs in this nanocomposite.

Further, the adsorption capacity of the sample was compared with that of Fe_3_O_4_ alone. Hakim *et al*. have already performed the CO_2_ adsorption-desorption studies of various types of iron oxides. They reported the adsorption capacity of 0.027 mmol g^−1^ at 25 °C and 1 bar pressure by Fe_3_O_4_ that had a surface area of 4.56 m^2^ g^−1^ ^30^. When compared with the individual capacity of NPC (2 mmol g^−1^) and Fe_3_O_4_ (0.027 mmol g^−1^) at 25 °C and 1 bar pressure, Fe_3_O_4_/NPC (4.25 mmol g^−1^) has shown a comparatively high adsorption capacity that might be due to the synergistic effect of Fe_3_O_4_ as well as the porous carbon support. Fe_3_O_4_/NPC shows high isosteric heat of adsorption values of about 38 kJ mol^−1^. The adsorption energies have been found to be much greater than 17.2 kJ mol^−1^, the enthalpy of liquefaction of CO_2_. These results imply that Fe_3_O_4_ particles enhance CO_2_ affinity and interaction strength mainly in the range of physisorption (Fig. 5(c and d))^31^. The calculation of the number of moles of gas adsorbed by the nanocomposite and the thermodynamic study have been discussed in SI. A comparison of the adsorption capacity of Fe_3_O_4_/NPC with some of the recent literature has been shown in Table S1.

From the adsorption isotherms, it can be inferred that adsorption capacity increases with increasing pressure but decreases with increasing temperature. From BET theory it is known that as pressure increases, multilayer adsorption takes place as more number of gas molecules are available per unit surface area of the sample^32^. This high adsorption capacity might be attributed to the good interaction between the gas molecules and the Fe_3_O_4_ decorated N doped porous carbon surface. Nitrogen doping and the presence of oxide nanoparticles account for the manifestation of a greater number of basic sites in the sample. Consequently, multilayer formation of CO_2_ gas at the pores of the nanocomposite might have occurred. Further, it can be seen that adsorption capacity decreases with increasing temperature^33^. This can be due to the increase in kinetic energy with the increase in temperature that helps the gas molecules to desorb faster from the surface active sites and pores. The steepness of the rise in the isotherm of the nanocomposite even at lower pressure indicates a stronger binding interaction of CO_2_ with the nanocomposite^34^. Surface modifications caused by the presence of Fe_3_O_4_ nanoparticles and nitrogen doping along with good porosity and surface chemistry has synergistically resulted in such a high adsorption capacity of Fe_3_O_4_/NPC. Table S1 shows a comparison table of the CO_2_ uptake at high pressure with reported literature.

### Electrochemical studies

#### Lithium-ion battery

**Figure 6:**
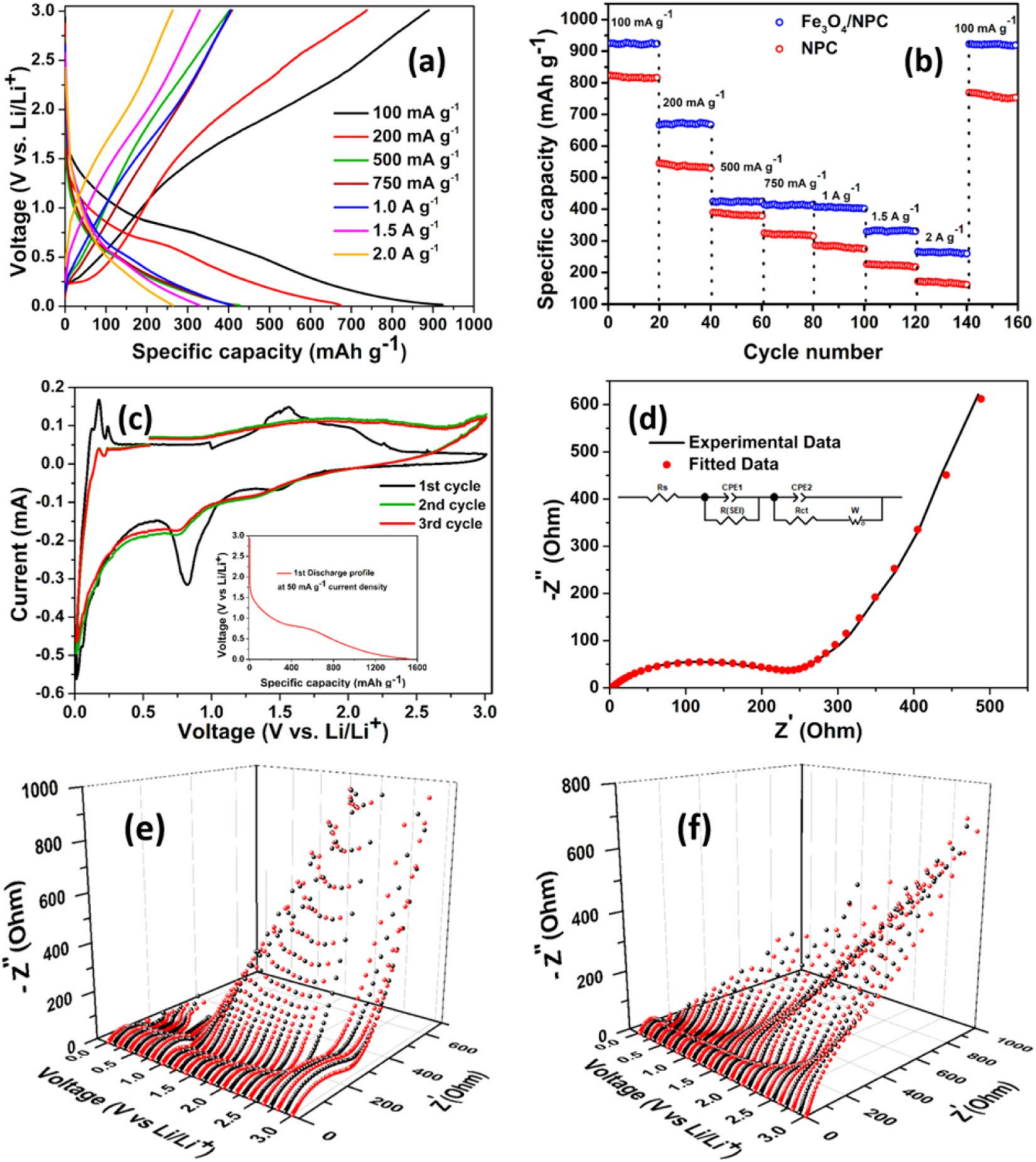
(**a**) Charge/discharge curves of Fe_3_O_4_/NPC anode at various current, (**b**) Rate performances of Fe_3_O_4_/NPC and NPC at various current densities, (**c**) First three cyclic voltammograms of Fe_3_O_4_/NPC at a scan rate of 0.1 mV sec^−1^ with first discharge profile (inset), (**d**) Electrochemical impedance profile in the discharged state after cycling (inset fitted circuit), (**e**) Discharging and (**f**) charging profiles of dynamic electrochemical impedance profile in the potential window of 0.01 to 3 V.

Half-cell measurements were carried out with Fe_3_O_4_/NPC as anode material for Lithium-ion battery. The galvanostatic charge-discharge profiles of 50^th^ cycle at various current densities are shown in Fig. 6(a). A discharge capacity of 930 mA h g^−1^ is observed at a current density of 100 mA g^−1^. With increasing current density, the discharge capacity has dropped down to 675 mA h g^−1^, 430 mA h g^−1^, 417 mA h g^−1^, 405 mA h g^−1^, 325 mA h g^−1^ and 263 mA h g^−1^ for current densities of 200 mA g^−1^, 500 mA g^−1^, 750 mA g^−1^, 1 A g^−1^, 1.5 A g^−1^ and 2 A g^−1^ respectively. The insignificant capacity loss with an increase in current density from 500 mA g^−1^ to 1 A g^−1^ suggests the synergetic effect of both insertion/de-insertion and conversion types of storage mechanism that has optimally enhanced the specific capacity without any significant change in the volume of the material (Fig. 6(b)). In literature, it is well reported that all metal oxide based anode materials suffer from large volume expansion/contraction upon lithiation/delithiation. For Fe_3_O_4_ the volume expansion can be up to 93% upon lithium intake^35^. So an anode consisting of only Fe_3_O_4_ suffers from large volume change which can cause severe cracking of the electrode and subsequent loss of electrical contact between individual particles, which in turn can result in severe capacity fading. In order to minimize the aforementioned issue, nitrogen doped porous carbon has been used as a support. Abundant mesopores not only promote the uninterrupted lithium intercalation/de-intercalation, but it also provide sufficient space for Fe_3_O_4_ particles to expand and contract without any significant change in the overall volume of the composite.

In order to compare with NPC, the anodic half-cell made of NPC has been cycled with similar current densities (Fig. S2(a)). The Galvanostatic charge-discharge profile of pure Fe_3_O_4_ is shown in Fig. S2(b). The characteristic plateau below 1.0 V indicates the interaction of Li^+^ ion with the electrolyte and subsequent formation of SEI layer can be observed only in the first discharge curve and disappears for further cycles Fig. 6(c) (inset)^36^. Unlike its Fe_3_O_4_ incorporated counterpart, NPC exhibits a significant capacity fading with an increase in current density from 500 mA g^−1^ to 1 A g^−1^. It can be concluded from the CV profile (Fig. S2(c)) that the contribution towards the specific capacity is only coming from the insertion/de-insertion mechanism in the porous network, as no redox peak can be seen in the CV profile. The broad reduction peak during the first cycle can be assigned to the SEI layer formation. The CV profile of Fe_3_O_4_/NPC exhibits strong evidence of stable SEI layer formation. Except for the first curve, all the charge-discharge cycles presented in Fig. 6(a) is taken after 50 cycles for each current density. No prominent change in the shape of the discharge curve implies superior cyclic stability of the anode material. Fe_3_O_4_/NPC shows excellent capacity retention of 83% whereas, in the case of NPC the retention dropped down to 72% after 100 cycles. Both the anode exhibit almost 100% coulombic efficiency (Fig. S2(d)). The comparison between the specific capacities obtained in the present work and previously reported literature has been shown in Table S2.

In order to have insight knowledge about the redox reactions occurring during charge-discharge, cyclic voltammetry (CV) was carried out in the potential range of 0.01 V–3.0 V (vs. Li/Li^+^) at a scan rate of 0.1 mV s^−1^. In Fig. 6(c), the first three cycles of CV measurement have been shown. In the first cycle, two pronounced reduction peaks at 1.48 V and 0.8 V along with a low-intensity kink at 1.0 V can be observed. Peaks at 1.48 V and 1.0 V corresponding to the two step reduction process from Fe^+3^/Fe^+2^ to Fe° which is in good agreement with literature^13,15,37–39^. The corresponding reactions are given as follows:

1 $${{\rm{Fe}}}_{{\rm{3}}}{{\rm{O}}}_{{\rm{4}}}+{{\rm{xLi}}}^{+}+{{\rm{xe}}}^{-}\leftrightarrow {{\rm{Li}}}_{{\rm{x}}}({{\rm{Fe}}}_{{\rm{3}}}{{\rm{O}}}_{{\rm{4}}})$$

2 $${{\rm{Li}}}_{{\rm{x}}}({{\rm{Fe}}}_{{\rm{3}}}{{\rm{O}}}_{{\rm{4}}})+(8-{\rm{x}}){{\rm{Li}}}^{+}+(8-{\rm{x}}){{\rm{e}}}^{-}\leftrightarrow {\rm{3Fe}}+{{\rm{4Li}}}_{{\rm{2}}}{\rm{O}}$$

The peak around 0.8 V can be assigned to the electrolyte decomposition at the electrode surface and subsequent formation of solid electrolyte interface (SEI) layer. The peak has been found to disappear in 2^nd^ and 3^rd^ cycles of the CV measurement indicating the formation of a stable SEI layer and an irreversible capacity loss. From the 2^nd^ cycle onwards, the CV pattern is reproducible and confirms the reversible electrochemical oxidation-reduction reactions (Fe^+3^/Fe^+2^ ↔ Fe°) arising from lithium extraction-insertions respectively. During the anodic sweep, two prominent peaks at 1.5 V and 1.9 V signify corresponding two-step oxidation process of Fe° to Fe^+3^/Fe^+2^ respectively^15,37^. Along with all the above-mentioned peaks, a pair of submerged redox peaks at 0.05 V and 0.21 V corresponds to lithium intercalation and de-intercalation in the porous carbon framework. The EIS spectra of the same anodic half-cell have been shown in Fig. 6(d) (details in SI).

In order to elucidate the dynamic variation of the impedance during charging and discharging, dynamical electrochemical impedance spectroscopy (DEIS) has been carried out with the assembled anodic half-cell. In the DEIS technique, the variable frequency response of an AC signal is superimposed with a DC voltage in the same potential scan of the anodic half-cell. The superimposed DC potential on the corresponding AC amplitude holds the electrochemical state of the cell to a complete stationary state, which is advantageous over EIS. In order to have an in-depth knowledge about the electrode reaction as a function of the state of charge (SOC), this technique is beneficial over EIS because it does not hold the potential at the requisite potential point leading to a non-stationary state^40^. Two sets of spectra have been recorded during charging and discharging process in the potential window of 0.01 V to 3.0 V at a regular potential interval of 0.1 V. During discharge, (Fig. 6(e)) the decrease in radius of the capacitive semicircle after 2.5 V indicates the reduction of the Fe_3_O_4_ to Li_x_(Fe_3_O_4_) and the associated charge transfer process. After 1.5 V the increase in the radius of semicircles describes the associated impedance due to the formation of the SEI layer, which makes the insertion of Li^+^ ion difficult. In the potential window of 0.01 V to 1.0 V, the diffusion tails of the spectra exhibit minimum impedance.

During charging (Fig. 6(f)) the semicircles at higher frequency range represent resistance associated with SEI layer formation. After 2.0 V, the diameter of the semicircles increases gradually indicating the increase in resistance during Li^+^ ion de-insertion process due to the formation of the thick SEI layer. The same behavior can be interpreted from the diffusion tails of the impedance spectra above 2.0 V as well. The impedance associated with the Li^+^ ion diffusion in the porous network gets hindered because of the formation of the SEI layer.

#### Oxygen Reduction Reaction (ORR)

**Figure 7:**
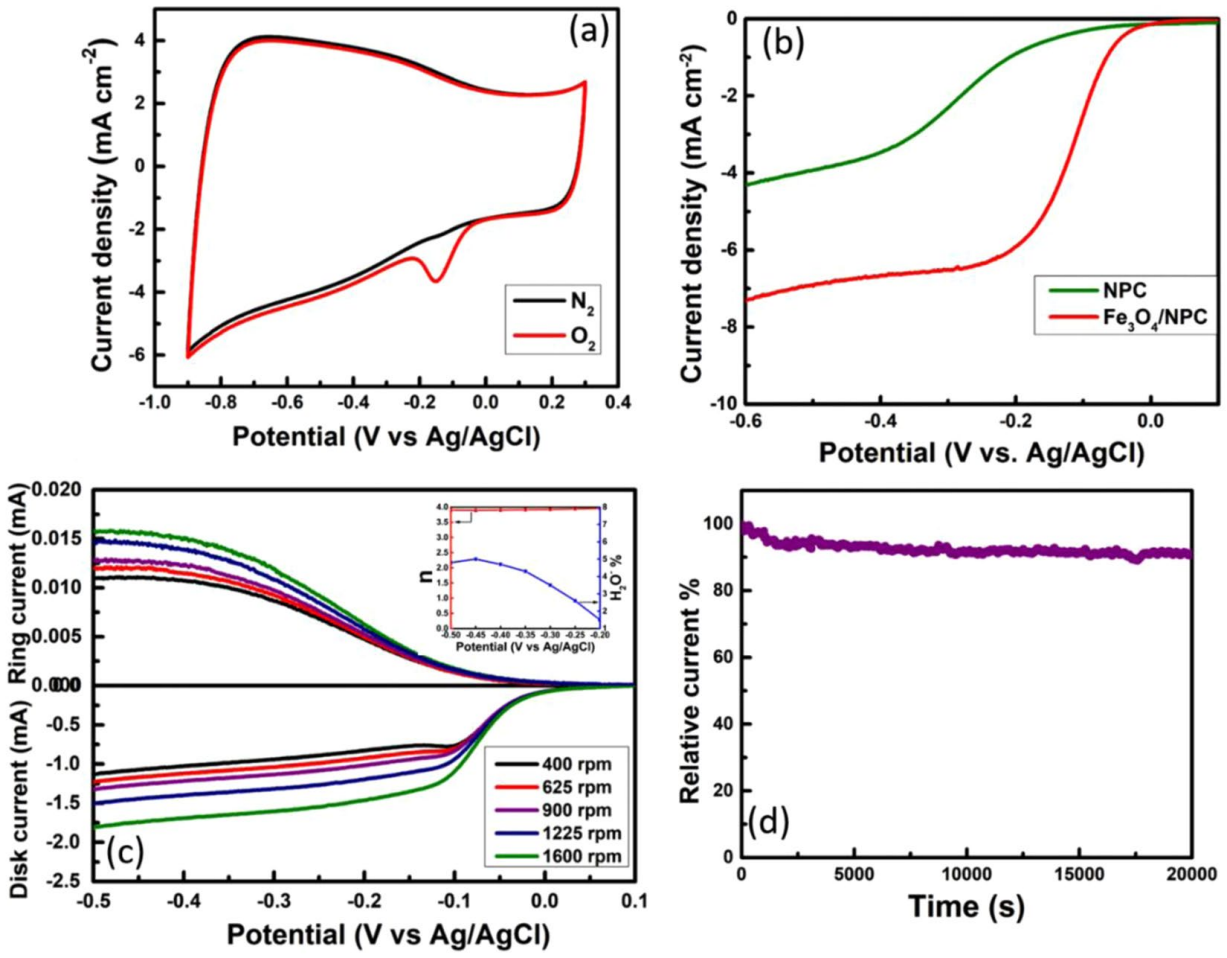
(**a**) Cyclic voltammograms of Fe_3_O_4_/NPC, (**b**) Rotating disk electrode voltammograms of NPC and Fe_3_O_4_/NPC, (**c**) Rotating ring-disk electrode voltammograms (inset number of electrons transferred (n) and peroxide ions produced at different potentials), (**d**) chronoamperogram at −0.15 V of Fe_3_O_4_/NPC.

The electrocatalytic activity of Fe_3_O_4_/NPC was investigated through cyclic voltammetry with a scan rate of 50 mV s^−1^ by saturating the 0.1 M KOH electrolyte with nitrogen and oxygen gas. Figure 7(a) represents the cyclic voltammograms of Fe_3_O_4_/NPC in nitrogen and oxygen saturated electrolyte. The cyclic voltammogram in the N_2_ saturated electrolyte exhibits double layer capacitance, while, in the oxygen-saturated electrolyte, a prominent oxygen reduction peak can be observed at −0.15 V.

In order to have an insight knowledge about the mechanism behind ORR, rotating disk electrode (RDE) technique was carried out. The linear sweep voltammograms were obtained in the potential window of −0.5 to 0.1 V at a scan rate of 10 mV s^−1^ in oxygen saturated 0.1 M KOH electrolyte at different rotation rates varying from 400 rpm to 1600 rpm as shown in Fig. S3. The limiting current density increases as the rotation rate increases due to the enhancement in the diffusion of electrolyte in the porous carbon material^41^. The onset potential and half-wave potential of Fe_3_O_4_/NPC at 1600 rpm are −0.03 V and −0.1 V respectively and the mass activity at −0.1 V is 35.8 mA mg^−1^. More the positive onset and half-wave potentials, higher is the catalytic activity. This is mainly due to the high specific surface area and mesoporous nature of NPC that enhances the density of active sites and mass transport of the ions, electrons, oxygen and water molecules through the catalyst^42^. It is well reported that pyridinic N present in the carbon network consists of a lone pair of electrons. Conjugation of pyridinic N lone pair of electrons with the graphene π system makes easy adsorption of O_2_ molecules on these sites. On the other hand, graphitic N in the NPC network facilitates the electron transfer from the carbon host to the oxygen anti-bonding orbitals^43,44^. Yousung Jung *et al*. have reported that graphitic N becomes pyridinic N by opening the cyclic C-N ring after electron and proton transfer reaction and has shown that both pyridinic N and graphitic N can act as active sites for ORR^45^. Hence the presence of pyridinic N and graphitic N in NPC network of Fe_3_O_4_/NPC enhances the limiting current density and improves the onset potential of ORR^46^. Herein, we have compared the onset and half-wave potential of Fe_3_O_4_/NPC with that of few reported Fe based nitrogen doped porous carbon catalyst (Table S3) and with commercial Pt/C catalyst (Table S4). Moreover, the ORR activity of NPC has been investigated in the same condition as Fe_3_O_4_/NPC and shown in Fig. S4(a,b). The onset and half-wave potentials of NPC are at −0.1 V and −0.24 V respectively which are even more negative than Fe_3_O_4_/NPC as shown in Fig. 7(b).

Effects of mass transfer on the kinetics of ORR along with the number of electrons transferred were further studied from the polarization curves of Fe_3_O_4_/NPC shown in Fig. S3. The Koutecky-Levich (K-L) plots (J^−1^ vs ω^−1/2^) were obtained from polarization curves at various rotation rates as shown in Fig. S3 inset. These K-L plots show linearity with a nearly constant slope over the potential range of −0.45 V to −0.3 V. This suggests that the ORR process follows the first order reaction kinetics^47^. The ORR in alkaline medium takes place in two ways: i) two-electron pathway which leads to the formation of peroxide ion (HO_2_^−^) as an intermediate (Eqs. 3 and 4) and ii) four-electron pathway where oxygen molecule will be completely reduced to OH^−^ ions (Eq. 5)^48^, which is the most favorable reaction for fuel cell and gives rise to higher current densities.

3 $${{\rm{O}}}_{{\rm{2}}}+{{\rm{H}}}_{{\rm{2}}}{\rm{O}}+{{\rm{2e}}}^{-}\leftrightarrow {{{\rm{HO}}}_{{\rm{2}}}}^{-}+{{\rm{OH}}}^{-};\,{{\rm{E}}}^{{\rm{0}}}=-\,{\rm{0.076}}\,{\rm{V}}\,{\rm{vs}}\,{\rm{SHE}}$$

4 $${{{\rm{HO}}}_{{\rm{2}}}}^{-}+{{\rm{H}}}_{{\rm{2}}}{\rm{O}}+{{\rm{2e}}}^{-}\leftrightarrow {{\rm{3OH}}}^{-};\,{{\rm{E}}}^{{\rm{0}}}={\rm{0.878}}\,{\rm{V}}\,{\rm{vs}}\,{\rm{SHE}}$$

5 $${{\rm{O}}}_{{\rm{2}}}+{{\rm{2H}}}_{{\rm{2}}}{\rm{O}}+{{\rm{4e}}}^{-}\leftrightarrow {{\rm{4OH}}}^{-};\,{{\rm{E}}}^{{\rm{0}}}={\rm{0.401}}\,{\rm{V}}\,{\rm{vs}}\,{\rm{SHE}}$$

In order to confirm the presence of peroxide intermediate, rotating ring-disk electrode (RRDE) (Fig. 7(c)) measurement was performed in O_2_ dissolved 0.1 M KOH electrolyte at a scan rate of 10 mV s^−1^ with different rotation rates (400, 625, 900, 1225 and 1600 rpm) by applying suitable ring potential. The number of electrons transferred (n) per oxygen molecule reduction and the percentage of peroxide ion formation is calculated from following equations (Eqs. 6 and 7):

6 $$n=4\times \frac{{I}_{d}}{{I}_{d}+\frac{{I}_{r}}{N}}$$

7 $$ \% H{O}_{2}^{-}=200\times \frac{\frac{{I}_{r}}{N}}{{I}_{d}+\frac{{I}_{r}}{N}}$$

where I_d_ is the disk current, I_r_ is the ring current and N is collection efficiency of Pt ring (37% for Pine instruments RRDE electrode). The calculated values of *n* and the percentage of HO_2_^−^ formed at different potentials varying from −0.5 V to −0.2 V is shown in Fig. 7(c) inset. The percentage of HO_2_^−^ varies from 5% to 1.5%, which indicates that the intermediate product formed is very less and mostly the reaction takes place in the four-electron pathway. Feng and Mullen *et al*. have reported the dependence of the peroxide ion yield on different loading of Fe_3_O_4_ for Fe_3_O_4_/N-GAs. They have shown that by increasing the Fe_3_O_4_ loading from 4.1 to 46.2 wt %, the yield of HO_2_^−^ decreased and with around 20 wt% of Fe_3_O_4_ they got approximately 48% of peroxide^49^. Whereas, in the present work, Fe_3_O_4_/NPC with 20 wt % of Fe_3_O_4_ loading showed 12 folds lesser HO_2_^−^ yield. The low yield of HO_2_^−^, the improvement in the onset and half-wave potential and the four-electron pathway of ORR for Fe_3_O_4_/NPC is due to the presence of pyridinic N and graphitic N sites^46,47,50^. The number of electrons transferred (*n*) per oxygen molecule reduction and the percentage of peroxide ion formation for NPC catalyst is shown in Fig. S4(c,d).

The durability of Fe_3_O_4_/NPC was tested through chronoamperometry technique at −0.25 V (ORR peak potential from CV) in O_2_ saturated 0.1 M KOH electrolyte at 1600 rpm as shown in Fig. 7(d). The Fe_3_O_4_/NPC shows good stability up to 20,000 sec with 91% of current retention. The better durability of Fe_3_O_4_/NPC is because of the active sites in Fe_3_O_4_/NPC that can promote the ORR with less overpotential and the mesopores that can facilitate the mass transport of ions. So the synergistic effect of Fe_3_O_4_ nanoparticles along with the highly porous nitrogen-doped carbon can show excellent catalytic activity towards ORR.

## Conclusions

In summary, we have synthesized an environment friendly nitrogen doped porous carbon-metal oxide composite with a high specific surface area. The mechanism of the pore formation along with the coprecipitation method to synthesize metal oxide nanoparticles has been discussed. To the best of our knowledge, there are no such reports available on such a multifunctional material being used for CO_2_ capture as well as for ORR catalyst and lithium-ion storage simultaneously. The as-prepared nanocomposite has shown an excellent CO_2_ uptake capacity (40.5 mmol g^−1^ at 25 °C and 20 bar pressure), a maximum reversible specific capacity of 930 mA h g^−1^ at 100 mA g^−1^ current density as an anode material in lithium-ion battery and superior catalytic activity as Pt-free catalyst in alkaline medium with 12 fold decrement in peroxide yield. Largely, we can conclude that nitrogen doped porous carbon with metal oxide nanoparticles decorated on them can be considered as a versatile nanocomposite that has promising applications in gas adsorption technology as well as in energy storage and conversion devices.

## Methods

### Synthesis of nitrogen-doped porous carbon (NPC)

Nitrogen-doped mesoporous carbon was synthesized using a simple thermal decomposition technique. At first, glucose (Sigma, ≥99.5%), sodium hydrogen carbonate (EMPLURA, 99%) and melamine (HIMEDIA, 99%) were mixed thoroughly in 1:1:1 ratio and carbonized at 800 °C for 2 h in a tubular furnace with a heating rate of 5 °C/min. Melamine acts as the nitrogen source and NaHCO_3_ acts as a porogen for creating porosity in the carbon framework. The furnace was allowed to cool down to room temperature and the sample was collected. The sample, thus obtained, was washed profusely with water and ethanol to remove any unreacted chemicals and sodium content in the sample. Then it was allowed to dry at 60 °C overnight and the final sample is named as NPC.

### Decoration of Fe_3_O_4_ nanoparticles over N doped porous carbon (Fe_3_O_4_/NPC)

Fe_3_O_4_ nanoparticles were synthesized using coprecipitation technique. 200 mg of porous carbon (NPC), thus obtained, was dispersed in deionized water for 6 h. Then two types of Fe salts were slowly added to this solution and allowed to stir overnight. FeSO_4_.7H_2_O (SISCO Research Laboratories, 99.5%) and FeCl_3_.6H_2_O (SDFCL, 98%) were taken in the stoichiometric ratio of 2:3 and added. Then the solution was heated up to 90 °C. Ammonia solution (25%) (RANKEM) was added drop-wise to the above solution until the pH became 9, which basically act as the reducing agent. The solution was stirred for another 30 min at 90 °C and then allowed to cool to room temperature. The black precipitate, thereby obtained, was washed repeatedly with DI water until it became neutral, filtered and dried. The obtained sample was again annealed at 700 °C for 1 h in an inert atmosphere to obtain pure Fe_3_O_4_ phase in the sample^51,52^ (Fig. 1(a)).

### Characterization techniques

The morphologies of the as-prepared samples were studied using Inspect F50 Scanning Electron Microscope from FEI and Technai G20 Transmission Electron Microscope. The elemental analyses were also determined using Inspect F50 instrument. The crystalline nature of the samples was characterized by Rigaku Smartlab X-Ray diffractometer with nickel-filtered Cu K_α_ radiation (λ = 0.154 nm) at 40 kV and 100 mA. Measurements were carried out from 10° to 80° with a step size of 0.02°. The specific surface area, total pore volume, and pore size analysis were done *via* nitrogen adsorption/desorption isotherms at liquid nitrogen temperature of 77 K using Micromeritics ASAP 2020 surface area and porosity analyzer using Brunauer-Emmett-Teller (BET) and Barrett-Joyner-Halanda (BJH) theories respectively. X-ray photoelectron spectroscopy was carried out using Specs X-ray photoelectron spectrometer with Mg K_α_ as the X-ray source and PHOIBOS 100MCD analyzer to find out the chemical composition of the samples. All the peaks are fitted using an iterated Shirley background. The line-shape used in every fit is a Doniach Sunjic form convoluted with a Gaussian/Lorentzian shape. Thermogravimetric analysis was carried on using SDT Q 600 from TA Instruments. The sample was heated from room temperature to 1000 °C in air atmosphere at a flow rate of 150 ml min^−1^ in order to find out the weight percent of Fe_3_O_4_ in the composite.

### Gas adsorption measurements

The adsorption isotherm studies were carried out for carbon dioxide gas at high pressure and temperature using volumetric analysis method in Sievert’s apparatus. Before each measurement, the samples were degassed at 250 °C in presence of high vacuum (10^−6^ mbar) for 3 h to remove moisture content and any dissolved impurities from the sample surface and to regenerate the adsorptive sites. 200 mg of sample was loaded in a quartz tube for each measurement. For CO_2_ adsorption studies, the temperature was varied from 25 °C to 100 °C and pressure was varied from 2 bar to 20 bar. A number of cycles were repeated to check the adsorption capacity and the results were found to be consistent.

### Electrode preparation for electrochemical measurements

The electrode slurry for battery operation was prepared by mixing as synthesized Fe_3_O_4_/NPC as the active material (75%) with polyvinylidene fluoride (PVDF) as binder (15%) and conductive carbon (10%) thoroughly in N-methyl-2-pyrrolidone (NMP) solvent. This slurry was uniformly coated over copper foil (with a thickness of 0.009 mm) using doctor blade method. Post-coating, the foil was dried at 120 °C for 8 hours in a vacuum oven. The electrodes with 12 mm diameter were cut from the coated foil. 2032 coin cells were assembled in an argon-filled glove box (mBraun; with controlled H_2_O and O_2_ < 0.1 ppm) using lithium foil as reference and counter electrode. Celgard 2400 microfiber dipped in electrolyte solution made of 1 (M) lithium hexafluorophosphate (LiPF_6_) dissolved in ethylene carbonate (EC) and dimethyl carbonate (DEC) (1:1 v/v) served as the separator. A Biologic SP-300 electrochemical workstation was used to estimate the electrochemical performances. The galvanostatic charge/discharge had been carried out in the potential range of 0.01 V to 3.0 V with current densities in the range of 100 mA h g^−1^ to 2 A h g^−1^. All impedance spectra were taken in the frequency range of 1 MHz to 10 mHz. All the measurements were carried out at room temperature.

Catalytic activity towards ORR was investigated using cyclic voltammetry data. The electrochemical measurements were taken using three-electrode system with Biologic SP-300 workstation and Rotating Electrode Speed Controller (Pine Research Instrumentation) was used to control the rotation rate of working electrode during RDE and RRDE measurements. A platinum wire was used as a counter electrode and Ag/AgCl dipped in 1(M) KCl was used as the reference electrode. A glassy carbon (GC) electrode (Pine Research Instrumentation, USA.) modified with electrocatalyst slurry served as the working electrode. The electrocatalyst slurry was prepared by taking the calculated amount of catalyst dispersed in DI water and Nafion solution (5 wt %). Then small amount slurry was drop-casted on the glassy carbon electrode (GCE). The electrolyte used for this measurement was 0.1 M KOH solution. Prior to use, the GCE surface was rubbed with alumina paste of particle size 1 μm, 0.3 μm, and 0.05 μm and the electrolyte was saturated with oxygen.

## Supplementary information

supp info
