# Supplementary material for: Green synthesis of nitrogen-doped self-assembled porous carbon-metal oxide composite towards energy and environmental applications
Source: Sci Rep. 2019 Mar 26;9:5187. doi: 10.1038/s41598-019-41700-5 (PMC6435743; doi:10.1038/s41598-019-41700-5)
Supplement: Supplementary file 1 — supp info [file 41598_2019_41700_MOESM1_ESM.docx]

**Supplementary Information for**

**Green synthesis of nitrogen-doped self-assembled porous carbon-metal oxide composite towards energy and environmental applications**

Arpita Ghosh^†^, Sreetama Ghosh^†^, Garapati Meenakshi Seshadhri^†^ and Sundara Ramaprabhu^*^^[[1]](#footnote-1)^

*Alternative Energy and Nanotechnology Laboratory (AENL), Nano Functional Materials Technology center (NFMTC), Department of Physics, Indian Institute of Technology Madras, Chennai 600036, India*

**Material Characterization**

**Figure 2A.c** represents the XRD pattern of Fe_3_O_4_/NPC. The corresponding diffraction peaks at 30.1 °, 35.4 °, 37.2 °, 43.2 °, 53.5 °, 57.5 °, 63.1°, 74.6° are indexed as (220), (311), (222), (400), (422), (511), (440) and (533) planes respectively of Fe_3_O_4_ (JCPDS 65-3107). The broad peak around 25 º corresponds to graphitic (002) plane of porous carbon. It can be clearly stated from **Figure 2A.c** that no mixed phases are present in the sample since after annealing the small intensity Fe_2_O_3_ peak also disappears forming pure Fe_3_O_4_/NPC.

In the TGA curve **(Figure 2B)** the initial weight loss of the sample at a temperature below 100 °C is due to the release of the moisture content of the sample. The material is then found to be stable up to 350 °C after which a rapid decomposition of the carbon content in the nanocomposite takes place which finally confirms the weight percentage of Fe_3_O_4_ in the nanocomposite to be around 20. TGA of NPC shows a weight loss of around 140 ºC due to dehydration and another major weight loss in the temperature range of 440-650 ºC that corresponds to the oxidation of porous carbon. The complete decomposition of NPC shows the purity of the sample.

**Thermodynamics of adsorption**

The isosteric heat of adsorption ( is a very important thermodynamic parameter which determines the interaction between the adsorbate gas molecules and the adsorbent material by measuring the change in enthalpy during adsorption. It can be calculated from the Clausius-Clapeyron equation, which is given by **equation 1**:


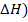

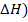


$\Delta H=R \left( \frac{\partial\ln P}{\partial\left( \frac{1}{T} \right)} \right)_{n}$ (1)


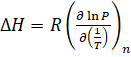


Since the isosteric heat of adsorption is temperature independent, the above equation can be written as **equation 2**:

$\ln P= \frac{\Delta H}{RT}+C$ (2)


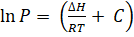


where P is the equilibrium pressure and R is the universal gas constant. C is the intercept of the isosteric curve with y-axis and T is the temperature of the isosteric points on the adsorption isotherm. is basically exothermic in nature and so is a negative quantity. It is measured from the slope of the adsorption isosteres by plotting *ln P* vs *1/T* (**Figure 5c**). So the absolute value of is basically called the isosteric heat of adsorption. The Fe_3_O_4_/NPC sample showed heat of adsorption of nearly 38 kJ mol^-1^ that indicates good interaction between adsorbed CO_2_ molecules and the sample. The adsorption energies have been found to be greater than 17.2 kJ mol^-1^ that is the enthalpy of liquefaction of CO_2_. Initially, during the increase in filling of pores by the gases, the enthalpy remains high which eventually decreases suggesting heterogeneity of the gas binding energies in the pores (**Figure 5d**).^1^


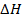

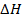

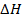

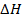


**Method to calculate the amount of CO_2_ adsorbed in the nanocomposite:**

The amount of gas adsorbed by the sample was measured by calculating the number of moles of gas before and after the adsorption process is given by **equation 3**:

${\Delta n}_{ads}= n_{i}- (n'+n'')$ (3)

where *n_i_* is the number of moles of gas present in volume *V_i_* having initial pressure *P_i_* where *V_i_ = V + V_d_*. *V* is the volume occupied by the standard volume and *V_d_* is the volume occupies by the SS tubes and connectors. *n'* denotes the number of moles of gas occupying volume *V_i_* at an equilibrium pressure *P_eq_* which is attained after the gas is allowed to equilibrate inside the sample cell. Finally, *n''* is the number of moles of gas present in sample cell volume *V_c_* at an equilibrium pressure *P_eq_*.

The corresponding values of *n_i_*, *n'* and *n''* can be calculated using the following equations (**equations 4-6**):

$ab{n_{i}}^{3}+ aV_{i}{n_{i}}^{2}+ \left( RT+ P_{i}b \right) V_{i}^{2}n_{i}- P_{i}V_{i}^{3}=0$ (4) $ab{n'}^{3}+ aV_{i}{n'}^{2}+ \left( RT+ P_{eq}b \right) V_{i}^{2}n' - P_{eq}V_{i}^{3}=0$ (5)

$ab{n''}^{3}+ aV_{i}{n''}^{2}+ \left( RT+ P_{eq}b \right) V_{c}^{2}n'' - P_{eq}V_{c}^{3}=0$ (6)

where T is the sample temperature and R = 8.314 J mol ^-1^ K ^-1^ is the universal gas constant. The values of the gas constants for CO_2_ are *a = 3.67 * 10^-1^ J m^3^ mol ^-2^* and *b = 4.32 * 10 ^-5^ m^3^ mol ^– 1^*.^2^

The XPS spectra of both NPC and Fe_3_O_4_ before and after coupling are presented in **Figure 2** and compared.


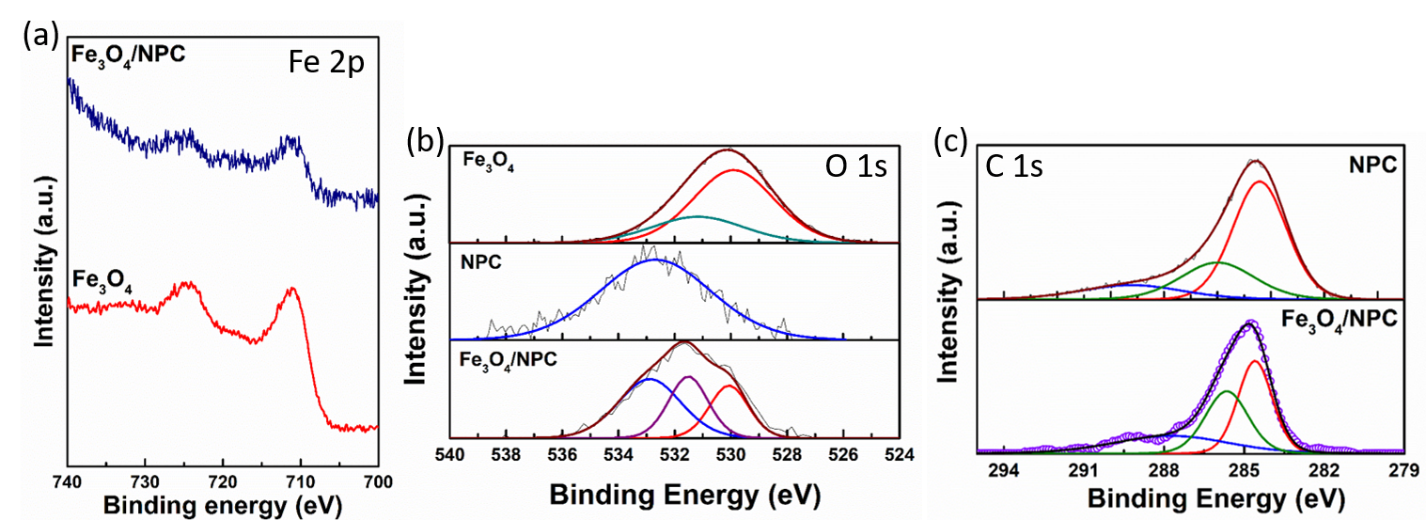


**Figure S1.** XPS curve fit of (a) Fe 2p, (b) O 1s and (c) C 1s spectra respectively before and after coupling.

In the high-resolution Fe 2p scan of Fe_3_O_4_, prominent peaks are seen at 711 and 724.4 eV that correspond to Fe 2p_3/2_ and Fe 2p_1/2_ respectively. Similar peak patterns are obtained in the Fe_3_O_4_/NPC composite as shown in **Figure S1 (a)** indicating the formation of Fe_3_O_4_ phase in the nanocomposite.^1^ **Figure S1 (b)** shows the O 1s spectra of Fe_3_O_4_ NPs, NPC and their composite. The deconvolution of the O 1s spectrum of Fe_3_O_4_ shows a prominent Fe‒O peak at 529.9 eV that corresponds to the anionic oxygen in Fe_3_O_4_.^1^ The other peak at 531.5 eV in Fe_3_O_4_ arises from the other oxygen-containing functional groups.^2^ The O 1s peaks of NPC consists of one peak arising from the oxygen functionalities such as C‒OH and/or C‒O‒C (532.6 eV).^3^ Now, the high-resolution O 1s core level spectra of the Fe_3_O_4_/NPC nanocomposite shows three peaks. A peak occurs at 529.9 that can be assigned as the Fe‒O peak coming from the Fe_3_O_4_ NPs present in the sample. Another peak at 532.5 eV occurs maybe from the NPC sample containing C‒OH and/or C‒O‒C functional groups. Another new broad peak occurs at the center at 531.7 eV in the nanocomposite. This peak might have been formed by the bonds between Fe_3_O_4_ and porous carbon and can be attributed to Fe‒O‒C bond between Fe_3_O_4_ and NPC.^1,4^ These findings can also be confirmed from variously reported literature that supports the formation of metal‒O‒C bonds having a positive shift of 1-3 eV from the metal‒O bonds.^1,4^  The C 1s spectra of NPC as shown in **Figure S1 (c)** can be deconvoluted into three components corresponding to C=C/C‒C (284.4 eV), C‒O/C‒N (286 eV) and C=O (289.2 eV).^3^ Interestingly, no peak shifts as such can be seen in the C 1s spectra of the nanocomposite, but it is seen that the peak intensities of carbon bonded to oxygen-containing functional groups have increased. This might be due to the contribution of the Fe_3_O_4_ NPs decorated on the surface of NPC.

Electrochemical impedance spectra (EIS) was taken in the above-mentioned frequency range after 50 cycles at 100 mA g^-1^ current density and fitted with the equivalent circuit shown in **Figure 6d**. The high frequency (~1 MHz) intercept at the real axis symbolizes the total ohmic resistance of 3.53 Ω, including the electrolyte resistance, contact resistance of all electronic connections.^3^ In **Figure 6d** a depressed semicircle at mid-frequency (~600 Hz) merged with a semicircle at low frequency (~25 Hz) which are the characteristics of Li^+^ diffusion through of solid electrolyte interface (SEI) and the charge transfer kinetics at the electrode-electrolyte interface respectively.^4^ The numerical values of resistances R_SEI_ and R_ct_, obtained from the fitted circuit (**Figure 6d inset**) are 22.2 Ω and 5.74 Ω respectively. The higher value of R_SEI_ suggests the formation of a comparatively thick and stable SEI layer. Lastly, the low frequency (~0.5 Hz) diffusion tail can be due to the solid state Warburg diffusion of Li^+^ ions into the porous carbon framework.^3^

In order to compare with NPC, the anodic half-cell made of NPC has been cycled with similar current densities. In comparison with Fe_3_O_4_/NPC (930 mA h g^-1^), the maximum discharge capacity obtained for NPC at 100 mA g^-1^ current density is around 670 mA h g^-1^. With an increasing current density, the discharge capacity has dropped down to 550 mA h g^-1^, 417 mA h g^-1^, 318 mA h g^-1^, 284 mA h g^-1^, 220 mA h g^-1^ and 170 mA h g^-1^ for current densities of 200 mA g^-1^, 500 mA g^-1^, 750 mA g^-1^, 1 A g^-1^, 1.5 A g^-1^ and 2 A g^-1^ respectively (**Figure S2 (a))**. Although Fe_3_O_4_ possesses a theoretical capacity of 926 mA h g^-1^, the volume expansion/contraction upon lithiation/de-lithiation severely affects the stability of the cell composed of only Fe_3_O_4_. The first 5 cycles of the mentioned cell have been shown in **Figure S2 (b)**, where significant capacity fading can be observed. Pore shrinkage during lithiation/de-lithiation leads to poor diffusion and mass transfer losses in NPC anode. The high volume expansion of Fe_3_O_4_, present within the pores, upon lithium intake, helps in reducing the pore shrinkage and restacking of the carbon framework, unlike pure NPC anode. This can be considered as a probable reason for enhanced rate capability as well as the stability of the composite anode over the porous carbon anode. Unlike its Fe_3_O_4_ incorporated counterpart, NPC exhibits a significant capacity fading with an increase in current density from 500 mA g^-1^ to 1 A g^-1^. It can be concluded from the CV profile (**Figure S2 (c))** that the contribution towards the specific capacity is only coming from the insertion/de-insertion mechanism in the porous network, as no redox peak can be seen in the CV profile. The broad reduction peak during the first cycle can be assigned to the SEI layer formation. Fe_3_O_4_/NPC shows excellent capacity retention of 83 % whereas, in the case of NPC the retention dropped down to 72 % after 100 cycles. Both the anode exhibit almost 100 % coulombic efficiency **(Figure S2 (d))**.

**
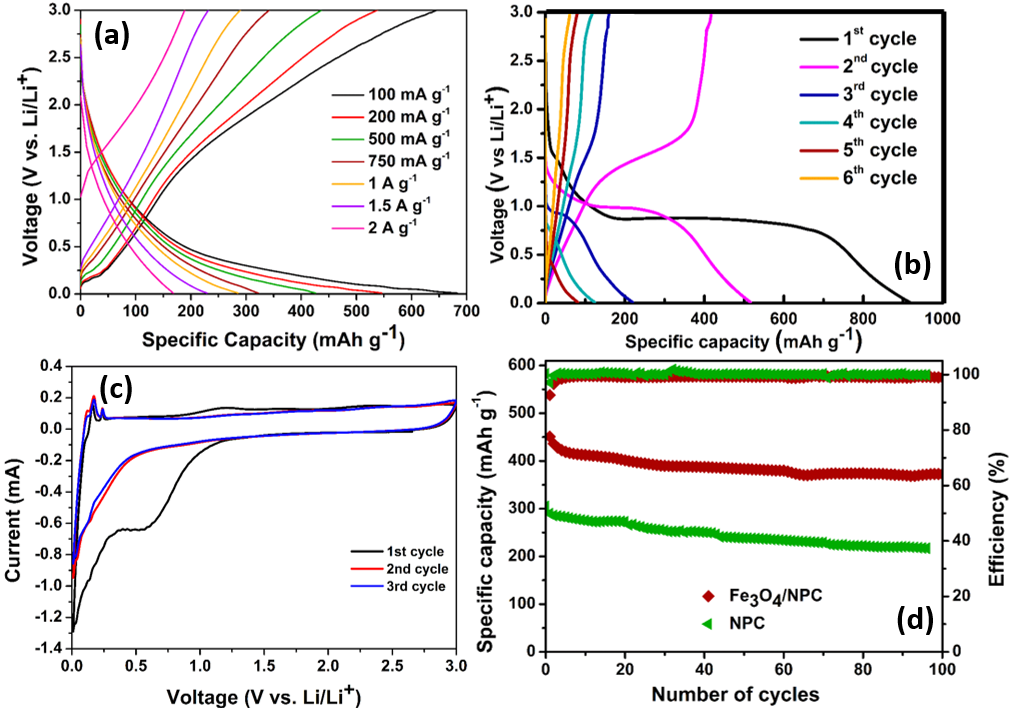
Figure S2.** (a) Galvanostatic charge-discharge profile of (a) NPC and (b) Fe_3_O_4_, (c) Cyclic voltammograms of NPC and (d) Capacity retention and coulombic efficiency of Fe_3_O_4_/NPC and NPC for 100 cycles taken at 1 A g^-1^ current density.


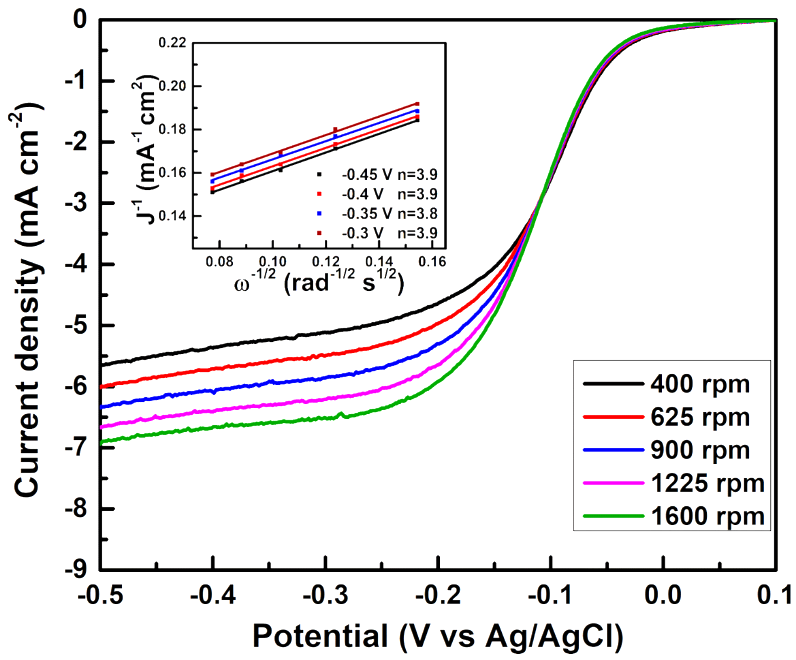


**Figure. S3** Rotating disk electrode voltammograms of Fe_3_O_4_/NPC at different rpm inset K-L plot.

The ORR activity of NPC has been studied in the same conditions as in Fe_3_O_4_/NPC (in 0.1 M KOH electrolyte and at room temperature). The cyclic voltammograms of NPC show an ORR reduction peak at -0.25 V **(Figure S4 (a))** whereas, for Fe_3_O_4_/NPC, the ORR peak was at -0.15 V (**Figure 7 (a)**). The linear sweep voltammograms were obtained in the potential window of -0.6 to 0.1 V at a scan rate of 10 mV s^-1^ in oxygen saturated 0.1M KOH electrolyte at different rotation rates varying from 400 rpm to 1600 rpm as shown in **Figure S4 (b).** The onset and half-wave potentials of NPC are at -0.1 and 0.24 V respectively which are even more negative than Fe_3_O_4_/NPC as shown in **Figure 7 (b)**. The number electrons transferred (n) per oxygen molecule reduction and the percentage of peroxide ion formation for NPC catalyst are also calculated using RRDE technique in the potential window of -0.6 to 0.1 V at a scan rate of 10 mV s^-1^ in oxygen saturated 0.1 M KOH electrolyte at different rotation rates varying from 400 rpm to 1600 rpm as shown in **Figure S4 (c)**. The NPC catalyst shows *n* value of approximately 3.3 and percentage of peroxide ion formation varies from 33 to 40 % in the potential window ranging from -0.6 to -0.1V **(Figure S4 (d))**. The incorporation of Fe_3_O_4_ nanoparticles, therefore, has enhanced the catalytic activity of NPC facilitating faster reaction kinetics in comparison to porous carbon.


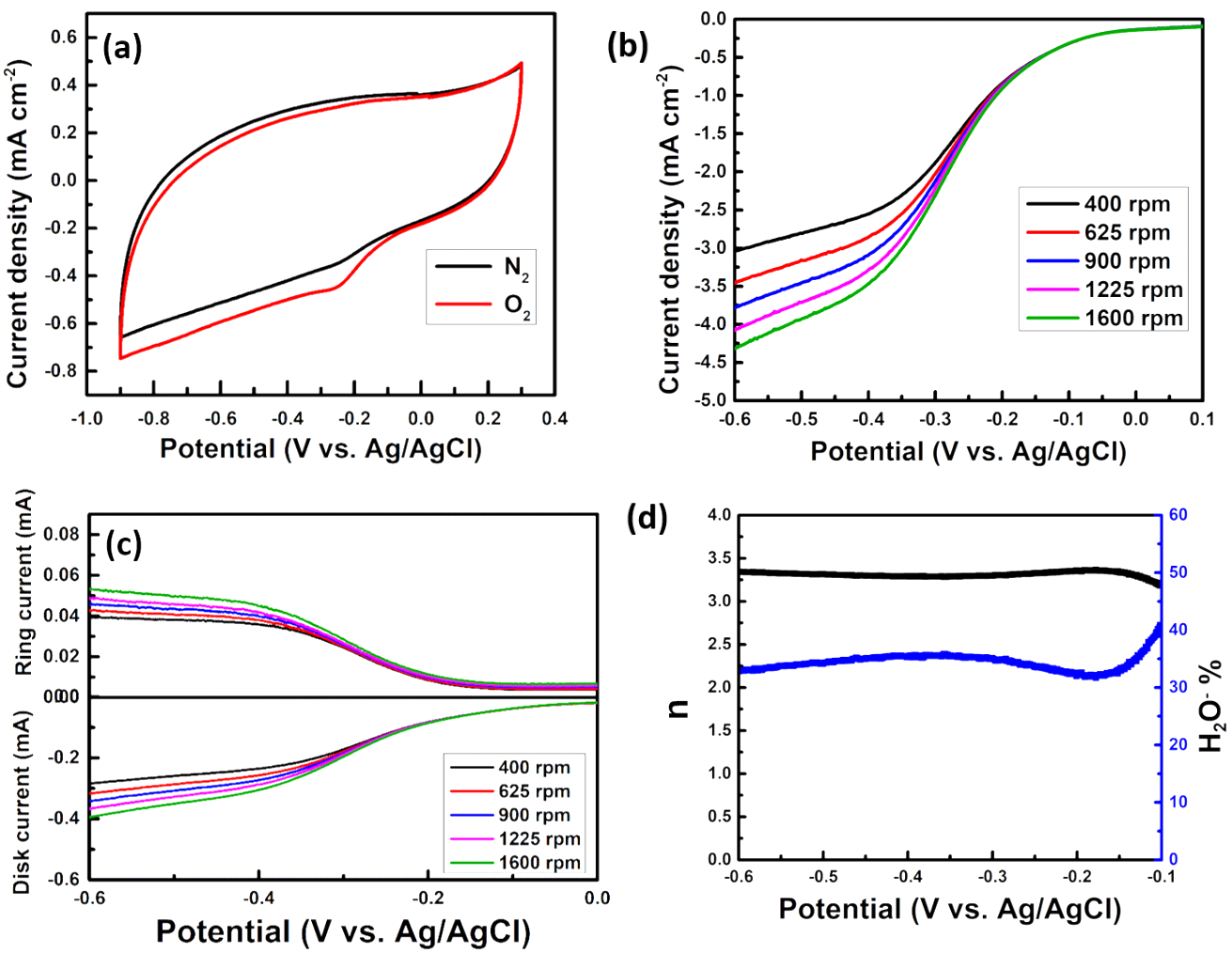


**Figure S4.** (a) Cyclic voltammograms, (b) Rotating disk electrode voltammograms, (c) Rotating ring-disk electrode voltammograms (d) number of electrons transferred (n) and peroxide ions produced at different potentials for NPC catalyst.

Koutecky-Levich (K-L) equation which is shown in **equation 7**: ^5^

$\frac{1}{J}=\frac{1}{J_{K}}+\frac{1}{J_{L}}= \frac{1}{J_{k}}+\frac{1}{B\omega^{1/2}}$ (7)

B = 0.62 n F (D_O2_)^2/3^ *v*^-1/6^ C_O2_ (8)

where J is the measured current density, J_K_ is the kinetic current density, J_L_ is the limiting current density, B is calculated from the slope of K-L plot (J^-1^ vs. ω^-1/2^) as shown in **equation 8**, F is Faraday’s constant (96485 C mol^-1^), D_O2_ is the diffusion constant of oxygen molecule in 0.1 M KOH solution (1.9 x 10^-5^ cm^2^ s^-1^), *v* is the kinematic viscosity of 0.1 M KOH solution (0.01 cm^2^ s^-1^) and C_O2_ is the bulk concentration of oxygen dissolved (1.2 x 10^-6^ mol cm^-3^).^6^

**Characterization of the samples after the test:**

In case of Li-ion battery, the cycled cell has been decrimped in argon atmosphere inside glove-box in a completely discharged state. The cycled electrode has been washed with the salt-free electrolyte and then subjected to XRD and XPS analysis. The XRD pattern of the cycled electrode is shown in **Figure S5.** Intense peaks at 43.36 ° and 50.51 ° confirm the presence of Li_2_CO_3_ (ICDD Reference No. 00-009-0359, 00-001-0996). The peaks at 44.49 ° and 74.21 ° signify the existence of Fe (ICDD Reference No. 00-001-1262) and Li_2_O (ICDD Reference No. 00-012-0254) respectively in the discharged state which is well consistent with the reaction mechanism mentioned in Eq. 2 in the main manuscript. Comparatively less intense peaks at 26.71 ° and 81. 99 ° is due to the presence of PVDF (ICDD Reference No. 00-042-1650) and LiOH (ICDD Reference No. 00-025-0486). The SEI layer formed during the 1^st^ discharge cycle mainly consists of Li_2_CO_3_, Li_2_O and LiOH. The high intensity of the Li_2_CO_3_ peaks suggests the formation of a stable SEI layer which is essential for the longevity of the cell.


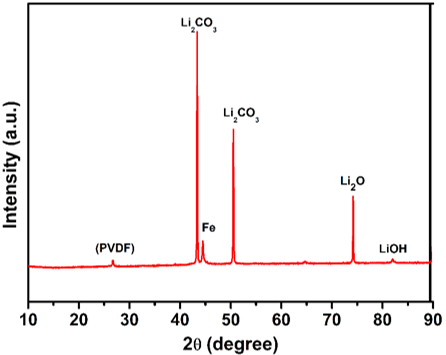


**Figure S5.** XRD pattern of the cycled anode (Fe_3_O_4_/NPC).

The aforementioned compositional analysis of the cycled electrode is well consistent with the XPS analysis shown in **Figure S6**. High-resolution XPS spectra of C 1s, O 1s and Fe 3p/Li 1s have been deconvoluted. Fe 3p and Li 1s have a substantial overlap, which makes it difficult to distinguish both the elements separately. C 1s has been deconvoluted into six components. Along with C=C, C–N (CO_x_) species additional peaks at 283.7 eV, 287.15 eV and 289.49 eV signifies the presence of Li–C, Li_2_CO_3_ and PVDF.^7^ O 1s spectra shows the presence of lithium alkoxide type species arising due to the degradation of EC (530.74 eV) along with Li_2_CO_3_ (530.37 eV).^7^ The main component at 55.5 eV for Fe 3p/Li 1s signifies the presence of Li_2_CO_3_ and lithium alkoxide.^7^

**
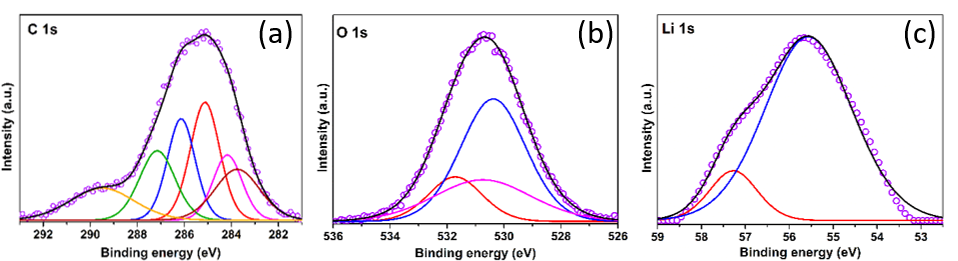
**

**Figure S6.** High resolution XPS spectra of (a) C 1s, (b) O 1s and (c) Li 1s taken for the cycled electrode.

From the SEM images (**Figure S7**) of the electrodes before and after cycling, the formation of a thick coating of the SEI layer over the porous carbon-metal oxide composite can be confirmed. Due to the formation of the SEI layer on the surface of the cycled electrode, the porous nature of the sample cannot be detected further. The EDAX analysis shows the presence of Fluorine which can be attributed to the formation of LiF during electrolyte decomposition or from the PVDF binder. The presence of Phosphorus can come from the composite Li_x_PF_y_O_z_ formed after cycling.^7^ Copper in EDAX is coming from the copper current collector.


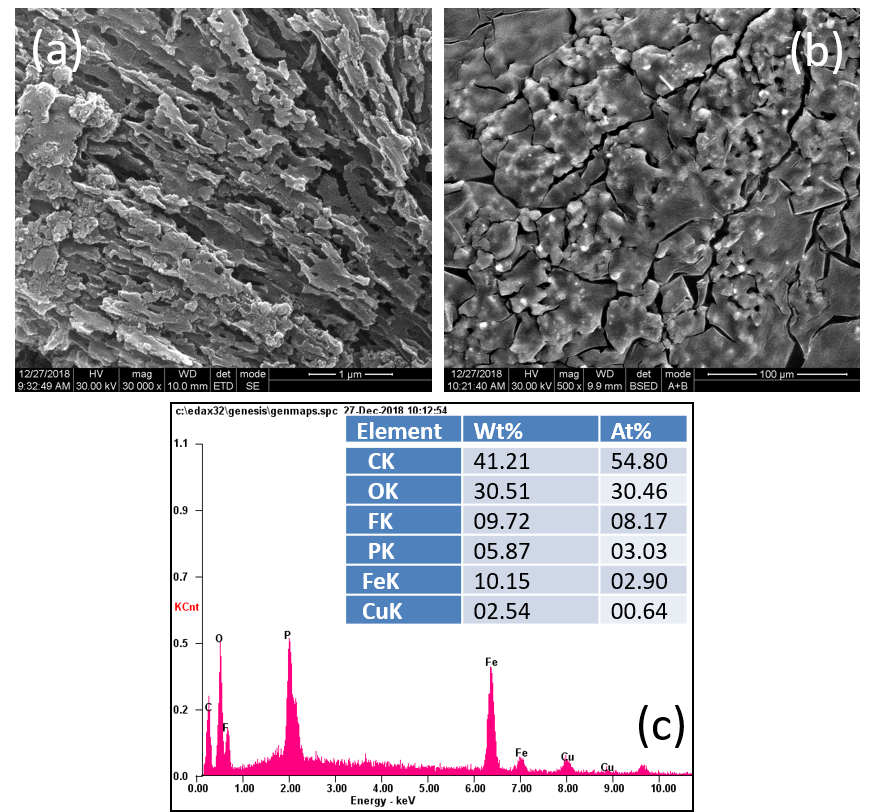


**Figure S7.** SEM images of the Fe_3_O_4_/NPC electrode (a) before cycling and (b) after cycling and (c) EDAX analysis of the electrode after cycling.

**Figure S8** represents the TEM image of Fe_3_O_4_/NPC sample after ORR durability test done using chronoamperometry (CA) technique. Fe_3_O_4_ nanoparticles decorated over NPC support after ORR durability test have been highlighted in red color circles. It is clearly visible from the TEM image that the size of the Fe_3_O_4_ nanoparticles has increased after the test when compared to Fe_3_O_4_/NPC sample before testing (as shown in **Figure 3d** in the main manuscript). This can be the reason for the decrement of current retention to 91% after 20,000 s of CA measurement. Even after the durability test, few Fe_3_O_4_ nanoparticles of smaller size and some pores in the NPC support can be observed from the TEM image. This signifies that the coalescence of Fe_3_O_4_ nanoparticles is slower when compared to Pt-based catalysts reported in literature^5,6^ which suggests that Fe_3_O_4_/NPC sample has good stability towards ORR.

**
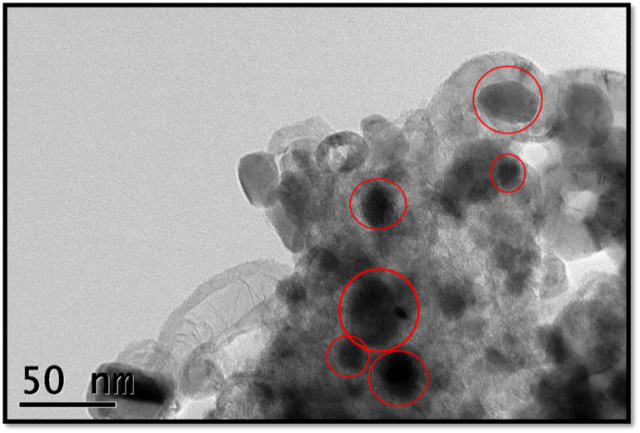
**

**Figure S8.** TEM image of Fe_3_O_4_/NPC after ORR durability test.

The FTIR transmittance spectra of Fe_3_O_4_/NPC are analyzed before and after CO_2_ adsorption. A peak appears at 589 cm^-1^ for both the spectra that can be attributed to Fe‒O bond confirming the existence of Fe_3_O_4_ in the composite.^8^ An intense band at 3420 cm^-1^ is associated with the stretching of O‒H bond present in the sample. Two new peaks appear in the sample after CO_2_ adsorption. A small peak at 2320 cm^-1^ can be as assigned to the asymmetric stretching of the adsorbed CO_2_ molecule due to physical adsorption. Besides, one more peak appears around 1636 cm^-1^ that can be attributed to bicarbonate formation as shown in **Figure S9**.^9^





**Figure S9.** FTIR absorbance spectra of Fe_3_O_4_/NMC before and after CO_2_ adsorption.

**Table S1.** CO_2_ uptake at high pressure for different reported porous carbon sorbents at 25 ºC.

| **Sample** | **Surface area (m^2^ g^-1^)** | **CO_2_ uptake**  **(mmol g^-1^)** | **References** |
| --- | --- | --- | --- |
| SD2600 | 1202 | 9.70 (20 bar) | ^7^ |
| a-GDC-1 | 2448 | 16.10 (20 bar) | ^8^ |
| PPy-500-2 | 1255 | 11.27 (20 bar) | ^9^ |
| Pan-600-3 | 1410 | 10.84 (20 bar) | ^9^ |
| rNPC | 1450 | 16.80 (30 bar) | ^10^ |
| KLB2 | 1122 | 11.90 (30 bar) | ^11^ |
| PPy-800-2 | 3230 | 21.01 (30 bar) | ^9^ |
| PPy-800-4 | 3450 | 22.10 (30 bar) | ^9^ |
| **Fe_3_O_4_/NPC** | **769** | **40.5 (20 bar)** | **Present work** |

**Table S2.** Specific capacity obtained at specified current density for Fe_3_O_4_ (or different metal oxide) based carbon composites from reported literature.

| **Sample** | **Weight % of metal oxide (wt %)** | **Specific capacity at specified current density (mA h g^-1^)** | **References** | |
| --- | --- | --- | --- | --- |
| Fe_3_O_4_ @C-3 | 56.5 | 700 (1 A g^-1^) | ^12^ |  |
| Fe_3_O_4_/Fe/Carbon | 46 | 500 (500 mA g^-1^) | ^13^ | |
| Fe_3_O_4_ NPs/GF | 76 | 470 (1 A g^-1^) | ^14^ | |
| Fe_3_O_4_-GNS | 38 | 650 (100 mA g^-1^) | ^15^ | |
| Hierarchical porous hollow nitrogen (N)-doped Fe_3_O_4_/C | Not mentioned | 400 (1 A g^-1^) | ^16^ | |
| Fe_3_O_4_@PCF | 60.8 | 700 (1 A g^-1^) | ^17^ | |
| **Fe_3_O_4_/NPC** | **20** | **405 (1 A g^-1^)** | **Present work** | |

**Table S3.** Comparison of ORR performance of Fe_3_O_4_/NPC in 0.1 M KOH electrolyte (potential referred to Ag/AgCl electrode) with all Fe based nitrogen doped porous carbon catalysts in literature.

| **Catalysts** | **Specific surface area (m^2^ g^-1^)** | **Onset Potential**  **(V)** | **Half-wave potential**  **(V)** | **References** |
| --- | --- | --- | --- | --- |
| Fe_3_O_4_/N-GAs | 100 | -0.19 | -0.3 | ^18^ |
| Fe_3_O_4_/N-C 900 | 210 | -0.05 | -0.18 | ^19^ |
| Fe/N/C HNSs-750 | 736 | -0.12 | -0.22 | ^6^ |
| 3DHP/Fe-N-C-900 | 906.3 | 0 | -0.15 | ^20^ |
| Fe_2_N@NPC-500  Fe-Mt-SS-C | 381.4  784.4 | -0.038  0.03 | -0.175  -0.09 | ^21^  ^22^ |
| **Fe_3_O_4_/NPC** | **769** | **-0.03** | **-0.1** | **Present work** |

**Table S4.** Comparison of onset potential of Fe_3_O_4_/NPC with commercial Pt/C (20 wt% Pt) in 0.1 M KOH electrolyte with Ag/AgCl as reference electrode.

| **Catalysts** | **Onset Potential (V)** | **Half wave potential (V)** | **References** |
| --- | --- | --- | --- |
| Pt/C | -0.02 | - | ^19^ |
| Pt/C | 0.03 | - | ^20^ |
| Pt/C | -0.02 | - | ^23^ |
| Pt/C | -0.05 | -0.33 | ^24^ |
| Pt/C  Pt/C | -  0.02 | -0.16  -0.18 | ^6^  ^22^ |
| **Fe_3_O_4_/NPC** | **-0.03** | **-0.1** | **Present work** |

**References:**

1. Sánchez-Sánchez, Á., Suárez-García, F., Martínez-Alonso, A. & Tascón, J. M. D. Influence of porous texture and surface chemistry on the CO_2_ adsorption capacity of porous carbons: Acidic and basic site interactions. *ACS Appl. Mater. Interfaces* **6,** 21237–21247 (2014).

2. Tamilarasan, P. & Ramaprabhu, S. Effect of partial exfoliation in carbon dioxide adsorption-desorption properties of carbon nanotubes Effect of partial exfoliation in carbon dioxide adsorption-desorption properties of carbon nanotubes. *J. Appl. Phys.* **116,** 124314 (2016).

3. Moss, P. L., Au, G., Plichta, E. J. & Zheng, J. P. An Electrical Circuit for Modeling the Dynamic Response of Li-Ion Polymer Batteries. *J. Electrochem. Soc.* **155,** A986 (2008).

4. Sahoo, M. & Ramaprabhu, S. Effect of wrinkles on electrochemical performance of multiwalled carbon nanotubes as anode material for Li ion battery. *Electrochim. Acta* **186,** 142–150 (2015).

5. Rana, M., Subramani, K., Sathish, M. & Gautam, U. K. Soya derived heteroatom doped carbon as a promising platform for oxygen reduction, supercapacitor and CO_2_ capture. *Carbon.* **114,** 679–689 (2017).

6. Zhou, D. *et al.* Fe/N/C hollow nanospheres by Fe(Ⅲ)-dopamine complexation-assisted one-pot doping as nonprecious-metal electrocatalysts for oxygen reduction. *Nanoscale* **7,** 1501–1509 (2015).

7. Balahmar, N., Al-Jumialy, A. & Mokaya, R. Biomass to porous carbon in one step: Directly activated biomass for high performance CO_2_ storage. *J. Mater. Chem. A* **5,** 12330–12339 (2017).

8. Ganesan, A. & Shaijumon, M. M. Activated graphene-derived porous carbon with exceptional gas adsorption properties. *Microporous Mesoporous Mater.* **220,** 21–27 (2016).

9. Ghosh, S. *et al.* Defining a performance map of porous carbon sorbents for high-pressure carbon dioxide uptake and carbon dioxide–methane selectivity. *J. Mater. Chem. A* **4,** 14739–14751 (2016).

10. Jalilov, A. S. *et al.* Asphalt-Derived High Surface Area Activated Porous Carbons for Carbon Dioxide Capture. *ACS Appl. Mater. Interfaces* **7,** 1376–1382 (2015).

11. Singh, G. *et al.* Single step synthesis of activated bio-carbons with a high surface area and their excellent CO_2_ adsorption capacity. *Carbon.* **116,** 448–455 (2017).

12. Zhang, X. *et al.* Fe_3_O_4_ @porous carbon hybrid as the anode material for a lithium-ion battery: performance optimization by composition and microstructure tailoring. *New J. Chem.* **39,** 3435–3443 (2015).

13. Zhao, X., Xia, D. & Zheng, K. Fe_3_O_4_/Fe/carbon composite and its application as anode material for lithium-ion batteries. *ACS Appl. Mater. Interfaces* **4,** 1350–6 (2012).

14. Zhang, N. *et al.* Electrostatically Assembled Magnetite Nanoparticles/Graphene Foam as a Binder-Free Anode for Lithium Ion Battery. *Langmuir* **33,** 8899–8905 (2017).

15. Wang, J. Z. *et al.* Graphene-encapsulated Fe_3_O_4_ nanoparticles with 3D laminated structure as superior anode in lithium ion batteries. *Chem. Eur. J.* **17,** 661–667 (2011).

16. Lim, H., Jung, B., Sun, Y. & Suh, K. Electrochimica Acta Hollow Fe_3_O_4_ microspheres as anode materials for lithium-ion batteries. **75,** 123–130 (2012).

17. Qin, X. *et al.* Fe_3_O_4_ nanoparticles encapsulated in electrospun porous carbon fibers with a compact shell as high-performance anode for lithium ion batteries. *Carbon.* **87,** 347–356 (2016).

18. Wu, Z., Yang, S., Sun, Y., Parvez, K. & Feng, X. 3D Nitrogen-Doped Graphene Aerogel-Supported Fe_3_O_4_ Nanoparticles as Efficient Electrocatalysts for the Oxygen Reduction Reaction. *J. Am. Chem. Soc.* **134,** 9082–9085 (2012).

19. Su, Y. *et al.* Enriched graphitic N-doped carbon-supported Fe_3_O_4_ nanoparticles as efficient electrocatalysts for oxygen reduction reaction. *J. Mater. Chem. A* **2,** 7281–7287 (2014).

20. Li, G. L. *et al.* Promotion of oxygen reduction performance by Fe_3_O_4_ nanoparticles support nitrogen-doped three dimensional meso/macroporous carbon based electrocatalyst. *Int. J. Hydrogen Energy* **42,** 4133–4145 (2017).

21. Huang, X. *et al.* In situ Fe_2_N@N-doped porous carbon hybrids as superior catalysts for oxygen reduction reaction. *Nanoscale* **9,** 8102–8106 (2017).

22. Chen, M. *et al.* Three-Dimensional Multi-Doped Porous Carbon/Graphene Derived from Sewage Sludge with Template-Assisted Fe-pillared Montmorillonite for Enhanced Oxygen Reduction Reaction. *Sci. Rep.* **7,** 1–10 (2017).

23. Qazzazie, D., Beckert, M., Mülhaupt, R., Yurchenko, O. & Urban, G. Modified graphene as electrocatalyst towards oxygen reduction reaction for fuel cells. *J. Phys. Conf. Ser.* **557,** (2014).

24. Molina-García, M. A. & Rees, N. V. Effect of catalyst carbon supports on the oxygen reduction reaction in alkaline media: a comparative study. *RSC Adv.* **6,** 94669–94681 (2016).

1. *Corresponding author. Tel: +91-44-22574862. E-mail: ramp@iitm.ac.in (Sundara Ramaprabhu).

   ^†^ Equally contributed. [↑](#footnote-ref-1)
